# Atomic Structure of IglD Demonstrates Its Role as a Component of the Baseplate Complex of the *Francisella* Type VI Secretion System

**DOI:** 10.1128/mbio.01277-22

**Published:** 2022-08-29

**Authors:** Xiaoyu Liu, Daniel L. Clemens, Bai-Yu Lee, Xue Yang, Z. Hong Zhou, Marcus A. Horwitz

**Affiliations:** a Department of Microbiology, Immunology and Molecular Genetics, University of California, Los Angeles (UCLA), Los Angeles, California, USA; b The California NanoSystems Institute (CNSI), UCLA, Los Angeles, California, USA; c Department of Medicine, UCLA, Los Angeles, California, USA; d State Key Laboratory of Medicinal Chemical Biology, Nankai University, Tianjin, China; University of California, Irvine

**Keywords:** type VI secretion system, baseplate complex, cryo-electron microscopy, *Francisella*, IglD, intracellular pathogen, contractile injection system

## Abstract

Francisella tularensis, a Tier 1 select agent of bioterrorism, contains a type VI secretion system (T6SS) encoded within the *Francisella* pathogenicity island (FPI), which is critical for its pathogenesis. Among the 18 proteins encoded by FPI is IglD, which is essential to *Francisella*’s intracellular growth and virulence, but neither its location within T6SS nor its functional role has been established. Here, we present the cryoEM structure of IglD from Francisella novicida and show that the *Francisella* IglD forms a homotrimer that is structurally homologous to the T6SS baseplate protein TssK in Escherichia coli. Each IglD monomer consists of an N-terminal β-sandwich domain, a 4-helix bundle domain, and a flexible C-terminal domain. While the overall folds of IglD and TssK are similar, the two structures differ in three aspects: the relative orientation between their β-sandwich and the 4-helix bundle domains; two insertion loops present in TssK’s β-sandwich domain; and, consequently, a lack of subunit-subunit interaction between insertion loops in the IglD trimer. Phylogenetic analysis indicates that IglD is genetically remote from the TssK orthologs in other T6SSs. While the other components of the *Francisella* baseplate are unknown, we conducted pulldown assays showing IglJ interacts with IglD and IglH, pointing to a model wherein IglD, IglH, and IglJ form the baseplate of the *Francisella* T6SS. Alanine substitution mutagenesis further established that IglD’s hydrophobic pocket in the N-terminal β-sandwich domain interacts with two loops of IglJ, reminiscent of the TssK-TssG interaction. These results form a framework for understanding the hitherto unexplored *Francisella* T6SS baseplate.

## INTRODUCTION

Francisella tularensis is a facultatively intracellular Gram-negative bacterium that causes the serious and potentially fatal zoonotic illness, tularemia (1, 2). As a zoonosis, its animal reservoirs include rabbits and aquatic rodents with biting insects, such as ticks and deer flies, acting as vectors, though possibly nonessential to its enzootic persistence (3). Humans acquire the disease via bites from infected insects or by contact with infected animals or materials from the infected animals (2). Because of its extraordinarily high infectivity (inhalation of as few as 10 bacteria can cause a serious infection (4, 5)) and mortality, especially when inhaled, F. tularensis is considered a potential bioterrorism agent and is classified as a Tier 1 select agent (6).

*Francisella* causes disease by replicating intracellularly within cells of the infected animals and its intracellular life cycle has been well studied (7–9). After uptake by a process termed “looping phagocytosis” (10), the bacterium initially resides within a phagosome that acquires only limited late endosomal/lysosomal markers (7, 8). Subsequently, the bacterium permeabilizes its phagosome and replicates extensively within the cytosol (7, 8). A cluster of genes encoded on an island called the *Francisella* pathogenicity island (FPI) is essential to the capacity of the bacterium to disrupt its phagosome and replicate intracellularly (11, 12). It is thought that the FPI encodes an atypical type 6 secretion system (T6SS) (13). T6SS resembles an inside-out contractile phage and injects toxins and effector molecules into target prokaryotic or eukaryotic cells (14). The canonical T6SS consists of a membrane complex that anchors the system to the bacterial membrane, a baseplate complex, and a metastable contractile sheath that, upon contraction, drives an inner tube tipped with a spike and effector proteins across the bacterial inner and outer membranes and into the target cell (15).

Because the level of homology with other T6SSs is low and some of the components of other T6SSs have not been identified in the FPI, the *Francisella* T6SS has been classified as a separate system (T6SS^ii^) (16). F. tularensis has three major subspecies of clinical relevance to humans: F. tularensis subsp. tularensis (type A, found only in North America), F. tularensis subsp. holarctica (type B, found both in North America and in Europe), and F. tularensis subsp. mediasiatica (found primarily in central Asia) (17). A closely related species, F. novicida (Fn), is often used as a model to study the *Francisella* T6SS because of several features: it is of low virulence; it has the same intracellular life cycle (9); its genome has 98% identity at the nucleotide level with F. tularensis subsp. tularensis and subsp. holarctica (18); and it is genetically more tractable because it has a single FPI rather than two as in F. tularensis. While the proteins encoded on the *Francisella* T6SS show little sequence homology with T6SSs of other bacteria, the structures that have been determined to date show strong structural homology with components of other T6SSs. For example, we determined the structure of the *Francisella* T6SS sheath by cryoEM, showing that it was composed of IglA/IglB heterodimers that form hexagonal rings, which stack via an interlaced array of beta-strands (19). The sheaths of the canonical T6SS (20) and R-pyocin (21) have been shown to have structurally homologous sheath structures with corresponding interlacing β-strand meshwork. We have shown (22) that the *Francisella* central spike complex consists of trimeric PdpA and VgrG that has both structural similarities to, and differences from, the central spike of the canonical T6SS and other contractile injection systems with known structures.

The precontraction T6SS baseplate encloses the central spike and serves as a scaffold upon which the sheath and tube are assembled. While the atomic structure of the baseplates of canonical T6SS (23), R-pyocin (24), and T4 phage (25) have been determined, neither the composition nor the structure of the *Francisella* T6SS baseplate is known. The FPI encoded protein IglD is essential for phagosome escape and intracellular growth (26–28), and virulence in animals (29–31), but its function and role in the *Francisella* T6SS have not been established. Here, we report the purification of IglD from Fn and the determination of its cryoEM structure at 3.0 Å resolution. We show that IgID forms homotrimers with structural homology to TssK of the canonical T6SS. We also demonstrate that IglD interacts with IglJ and IglH and suggest how they serve as components of the *Francisella* T6SS baseplate.

## RESULTS

### Purification and cryoEM structure of Francisella novicida IglD.

The *iglD* gene was deleted from Fn and expressed under the control of the F. tularensis live vaccine strain bacterioferritin promoter on a plasmid with a 3× FLAG tag. The results showed that the complemented strain was fully functional in phagosomal escape (Fig. S1). T6SS was induced by growing the bacteria in high KCl as described previously (19, 22). Analysis of the purified material by Coomassie blue staining (Fig. S2A) showed a single protein band at about 47 kDa that corresponded to the IglD immunoreactive band. In the final step of purification, IglD eluted on Sephacryl S200 gel filtration in the same position as sweet potato amylase (MW 200 kDa), indicating that it forms a multimeric complex (Fig. S2A). Western immunoblotting confirmed that the protein staining band corresponded with a single band of immunoreactive material (Fig. S2B).

10.1128/mbio.01277-22.1 FIG S1 Fn *iglD* deletion strain expressing FLAG-IglD from a plasmid is capable of phagosomal escape in macrophage-like THP-1 cells. (A) The ability of Fn to escape its phagosome in THP-1 cells was assessed by immunofluorescence staining after differential permeabilization with digitonin (to detect Fn that had escaped their phagosomes, green fluorescence) and after permeabilization with Triton X-100 (to detect all Fn, red fluorescence). Size bars: 10 μm. (B) Quantitation of phagosome escape by differential immunofluorescent staining indicated that T6SS *iglC* and *iglD* genes are essential for Fn phagosome escape in the macrophage-like THP-1 cells. Expression of FLAG epitope-tagged *iglD* gene from a plasmid complemented the growth defect in the strain with *iglD* deletion. Data shown are mean ± standard error of at least 4 determinations. Download FIG S1, JPG file, 0.3 MB. Copyright © 2022 Liu et al. 2022 Liu et al. https://creativecommons.org/licenses/by/4.0/ This content is distributed under the terms of the Creative Commons Attribution 4.0 International license.

10.1128/mbio.01277-22.2 FIG S2 (A) Coomassie blue stained SDS-PAGE gel shows a single band at 45 kDa of FLAG-IglD protein purified from F. novicida by Q-sepharose ion exchange chromatography, anti-FLAG affinity chromatography, and Sephacryl S200 gel filtration. Elution position of FLAG-IglD in comparison with molecular weight markers that were run separately. (B) IglD complexes purified by ion exchange, affinity chromatography, and gel filtration showed a single band on SYPRO Ruby stained SDS-PAGE (L1) and Western immunoblot (L2). (C) Motion-corrected cryoEM micrograph. (D) Representative 2D class averages of IglD particles. (E) Plot of the FSC as a function of the spatial frequency with the indicated resolution. (F) Euler angle distributions of IglD particles used for the final reconstruction. (G) CryoEM map after C3 symmetry expansion with IglD structure solved in this study fitting in it. The yellow density corresponds to the C-terminal domain. Download FIG S2, JPG file, 0.6 MB. Copyright © 2022 Liu et al. 2022 Liu et al. https://creativecommons.org/licenses/by/4.0/ This content is distributed under the terms of the Creative Commons Attribution 4.0 International license.

**FIG 1:**
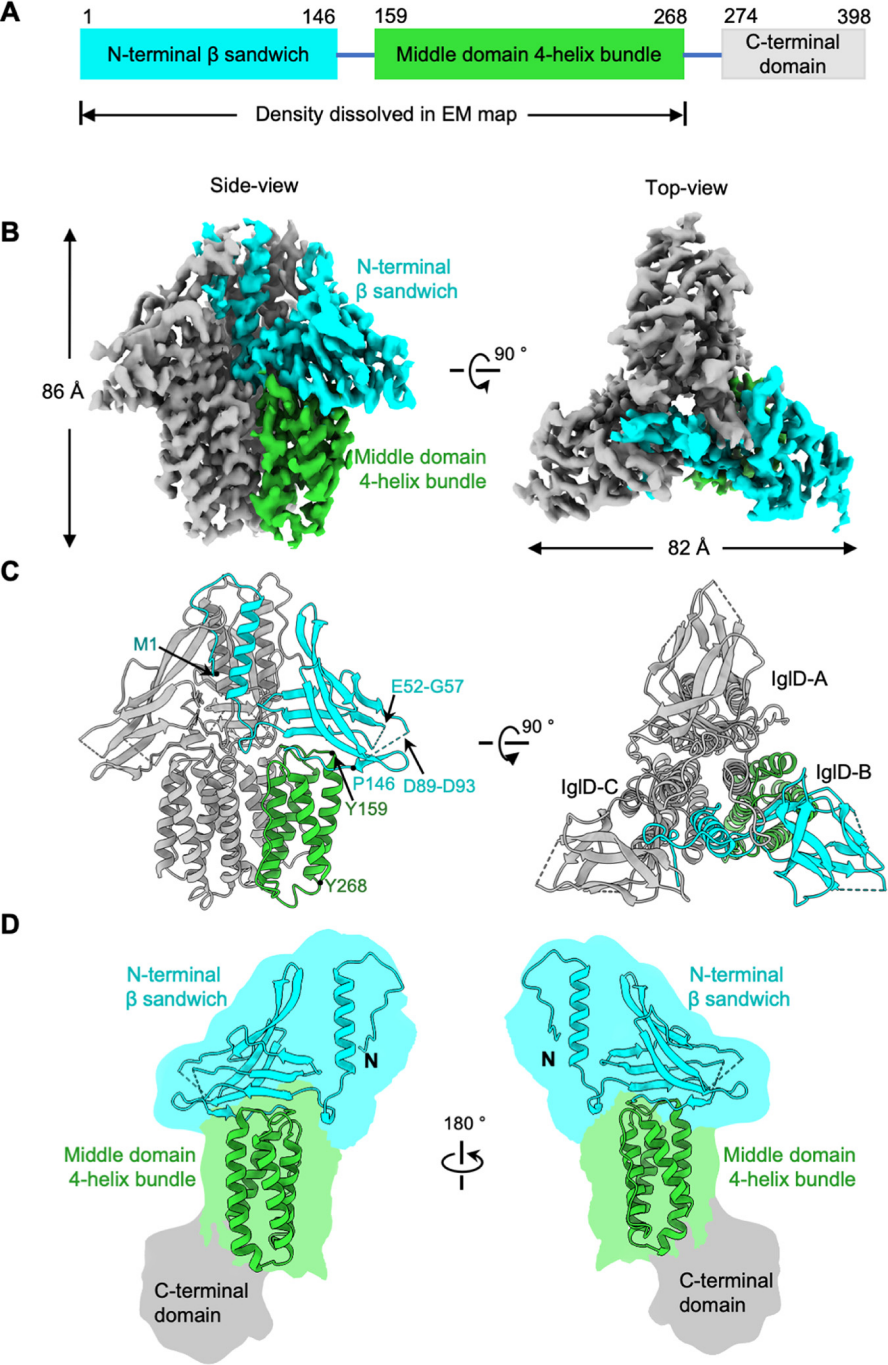
CryoEM structure of IglD. (A) Domain organization of Francisella novicida (Fn) IglD. The full-length protein consists of an N-terminal β sandwich (cyan), middle domain 4-helix bundle (green), and C-terminal domain (light gray). The C-terminal domain was not modeled. (B) Two different views of the cryoEM density map of IglD homotrimer. One protomer is represented with domains shown in the same color scheme as in A, the other two protomers are colored gray. (C) Two different views of IglD homotrimer structure. The structure is colored as in B. The three protomers are labeled as IglD-A, IglD-B, and IglD-C. (D) IglD monomer structure is shown in two different views. Domains are colored as in A. The N termini of the protein are labeled. Cartoon illustrations of full-length IglD monomer with C-terminal domain are shown based on the low-resolution map.

To obtain the structure of IglD, we recorded 2880 cryoEM images. We carried out a single-particle analysis (Fig. S2C to F and Fig. S3A) and generated a 3.0 Å-resolution reconstruction of sufficient quality (Fig. S3B) for the model building of the major parts of the protein. The structure was in a homotrimeric state with dimensions of 86 Å × 82 Å containing three IglD molecules (Fig. 1B), consistent with size exclusion chromatography. Among the 398 residues of the full-length protein, residues M1-Y268 were resolved in our map. Each subunit of the IglD trimer contained an N-terminal β sandwich (residues M1 to P146) and a middle 4-helix bundle domain (residues Y159 to Y268) (Fig. 1A). The N-terminal β sandwich started with a long α-helix followed by a β-sheet consisting of three anti-parallel β-strands. Between residues M1 to Y268, the cryoEM density corresponding to two connection loops (E52 to G57; D89 to D93) was visible but of insufficient quality to support atomic modeling (Fig. 1C). The C-terminal domain was not visible in our 3 Å map with C3 symmetry indicating the flexibility relative to the N-terminal part. After applying symmetry expansion and local classification, we obtained a map with extra density corresponding to the C-terminal domain (Fig. 1D and Fig. S2G). The extra densities were at a lower resolution and not symmetrical among the three IglD protomers, suggesting flexibility of this region.

10.1128/mbio.01277-22.3 FIG S3 (A) CryoEM data processing workflow. Schematic of the classification and refinement procedures used to generate the map obtained in this study (see Materials and Methods for details). (B) Representative density map of α helices and β strands. Download FIG S3, JPG file, 0.8 MB. Copyright © 2022 Liu et al. 2022 Liu et al. https://creativecommons.org/licenses/by/4.0/ This content is distributed under the terms of the Creative Commons Attribution 4.0 International license.

The trimeric structure of IglD resolves extensive intersubunit contacts (Fig. S4A), involving more than 30 residues in each protomer (Fig. S4B to D). Interactions were primarily through N-terminal helices forming a three-helix bundle and middle 4-helix bundle domain, further enhanced by domain-swapped-like interactions of the N-terminal loop with the adjacent protomer’s β sandwich. The interactions for trimerization were largely hydrophobic, with some salt bridges and hydrogen bonds involved.

10.1128/mbio.01277-22.4 FIG S4 (A to D) Trimer formation of IglD. (A) One protomer is shown as a ribbon diagram colored as in Fig. 1A, and the other two protomers are shown as the surface in gray. Key residues in trimerization are shown in sticks. (B to D) Enlarged view of different regions in (A). (E) Backbone hydrogen bonds between TssK-A loop1 and TssK-B loop2. Involved residues and distance are labeled. (F) Sequence alignment of F. novicida IglD (FnIglD) and EAEC TssK (EcTssK). The sequence alignment was done with Clustal Omega and displayed with ESPript 3. Secondary structures of FnIglD determined in this study are shown on top. Secondary structures labeled at the bottom were done according to EAEC TssK structural superposition (PDB accession no. 5M30). Download FIG S4, JPG file, 2.0 MB. Copyright © 2022 Liu et al. 2022 Liu et al. https://creativecommons.org/licenses/by/4.0/ This content is distributed under the terms of the Creative Commons Attribution 4.0 International license.

### IglD is homologous to E. coli TssK with similarities and differences.

**FIG 2:**
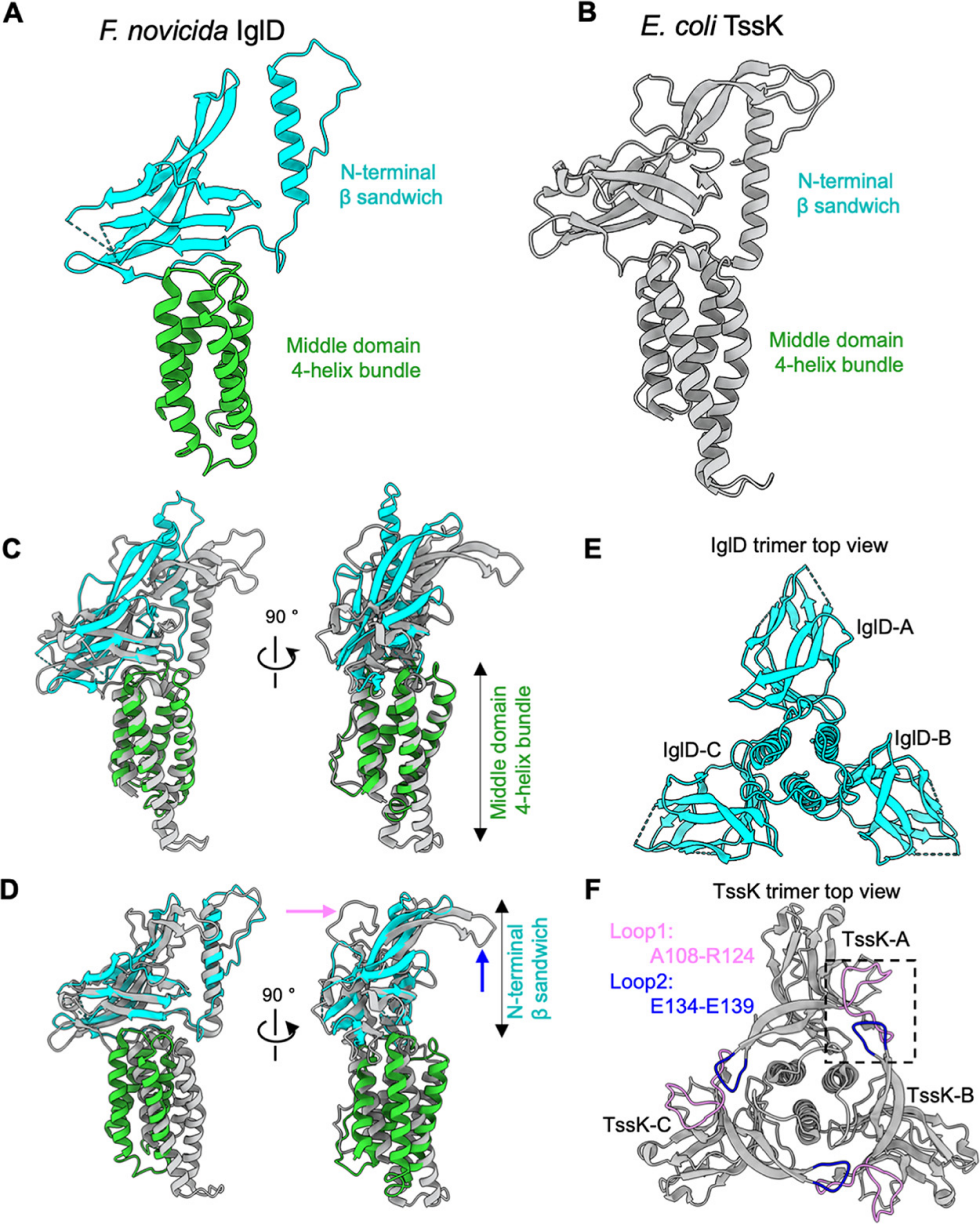
Structure comparison of IglD and TssK. (A) Structure of Fn IglD monomer. Domains are colored as in Fig. 1A. (B) Structure of EAEC TssK monomer (PDB accession no. 5MWN; Chain A). (C) Superposition of Fn IglD (color) and EAEC TssK (gray) monomers by 4-helix bundle domain. (D) Superposition of Fn IglD (color) and EAEC TssK (gray) monomers by N-terminal β-sandwich domain. The two arrows point to two insertion loops in TssK compared with IglD. The magenta one points to loop1 (A108-R124), the blue one points to loop2 (E134-E139). (E) IglD trimer top view, 4-helix bundle domain was omitted for clarity. The three protomers are labeled as IglD-A, IglD-B, and IglD-C. (F) TssK trimer top view, 4-helix bundle domain was omitted for clarity. The three protomers are labeled as TssK-A, TssK-B, and TssK-C. Loop1 in each protomer is colored magenta, and loop2 in each protomer is colored blue. The boxed region shows loop1 from TssK-A and loop2 from TssK-B.

Our results demonstrate that the *Francisella* IglD is structurally homologous to the canonical T6SS baseplate protein TssK (Fig. 2A and B). While the sequence identity and similarity between IglD and TssK (E. coli) are only 18.8% and 33.9%, respectively (Fig. S4F), the secondary structures (Fig. S4F) and the 3D structures of the two proteins are similar (Fig. 2A and B). Both IglD and TssK are homotrimers, containing an N-terminal β sandwich, middle 4-helix bundle domain, and C-terminal domain. By superimposing the IglD protomer structure onto TssK (Fig. 2C), they are automatically aligned by the 4-helix bundle domain based on maximum matching. It is apparent that the 4-helix bundle domain aligns well, while the N-terminal β sandwiches do not match even though both are composed of N-terminal helices and β strands. When we intentionally align the two structures by N-terminal β sandwiches, the two 4-helix bundle domains shift away from each other (Fig. 2D). These comparisons show that the relative orientation between their β-sandwich and the 4-helix bundle domains is different even though the overall folds of IglD and TssK are similar. Looking closely at the alignment of N-terminal β sandwiches, we found two loops (loop1: A108 to R124, loop2: E134 to E139) protrude in TssK compared to IglD (Fig. 2D). In the IglD trimer, the three protomers separate as three individual lobes (Fig. 2E). While in TssK trimer, a ring-like structure is formed mediated by intersubunit loop interactions mainly by hydrogen bonds (Fig. 2F and Fig. S4E). In the published TssK structures with a C-terminal domain, either by crystallography or cryoEM (23, 32), not all the C-terminal domains of three protomers can be traced, consistent with our assumption that the C-terminal domain was flexible. The C-terminal domain of TssK was shown to interact with the cytoplasmic domains of membrane complex (TssL and TssM) in T6SS. The flexibility of the C-terminal domain may provide a flexible link to maintain the anchorage of the baseplate to the membrane complex.

The DeepMind “AlphaFold2” artificial intelligence program has been employed to predict protein folding (33). After we obtained the structure of IglD, we sought to compare the model predicted by this program with the experimentally derived structure. We ran AlphaFold2 on google Colab (34) requesting the prediction of IglD as a monomer (Fig. S5A). The predicted structure consisted of an N-terminal β sandwich, middle 4-helix bundle domain, and C-terminal domain as with the experimentally derived structure. The superimposition of the two structures showed that they were not the same (Fig. S5B). We then divided the structure into 3 rigid domains and superimposed them individually. The N-terminal β sandwich, aside from the N-terminal helix, aligns very well with a root mean square deviation (RMSD) of 1.9 Å on 96 residues (Fig. S5C). For the 4-helix bundle domain of the two structures (Fig. S5D), the RMSD was 1.2 Å on 110 residues. Comparison of the experimental and predicted protomer structures revealed that the structures of each globular domain are highly similar even though the overall architectures are not the same. The C-terminal domain predicted by AlphaFold2 (Fig. S5E) is an assembly of two β sheets, one with five antiparallel β strands and the other with three. We then used AlphaFold2 to predict IglD as a homotrimer (Fig. S5F), which we had already established experimentally. The predicted trimer structure was unsatisfactory mainly because it lost the N-terminal helix trimerization and the domain-swapped interaction between protomers (Fig. S5F).

10.1128/mbio.01277-22.5 FIG S5 (A) Experimental and AlphaFold2 prediction of IglD monomer. AlphaFold2 model is colored according to pLDDT (predicted lDDT-Cα), a per-residue measure of local confidence. (B) Superposition of the predicted (gray) and experimental (cyan and green) IglD monomer structures. (C) Superposition of the predicted (gray) and experimental (cyan) N-terminal β sandwich. (D) Superposition of the predicted (gray) and experimental (green) 4-helix bundle domain. (E) Prediction of C-terminal domain. (F) AlphaFold2 prediction of IglD homotrimer. Three protomers are colored in magenta, yellow and gray, respectively. The boxed figure is colored according to pLDDT. Download FIG S5, JPG file, 0.8 MB. Copyright © 2022 Liu et al. 2022 Liu et al. https://creativecommons.org/licenses/by/4.0/ This content is distributed under the terms of the Creative Commons Attribution 4.0 International license.

### Identification of *Francisella* baseplate proteins by affinity pulldown and mass spectrometry.

**FIG 3:**
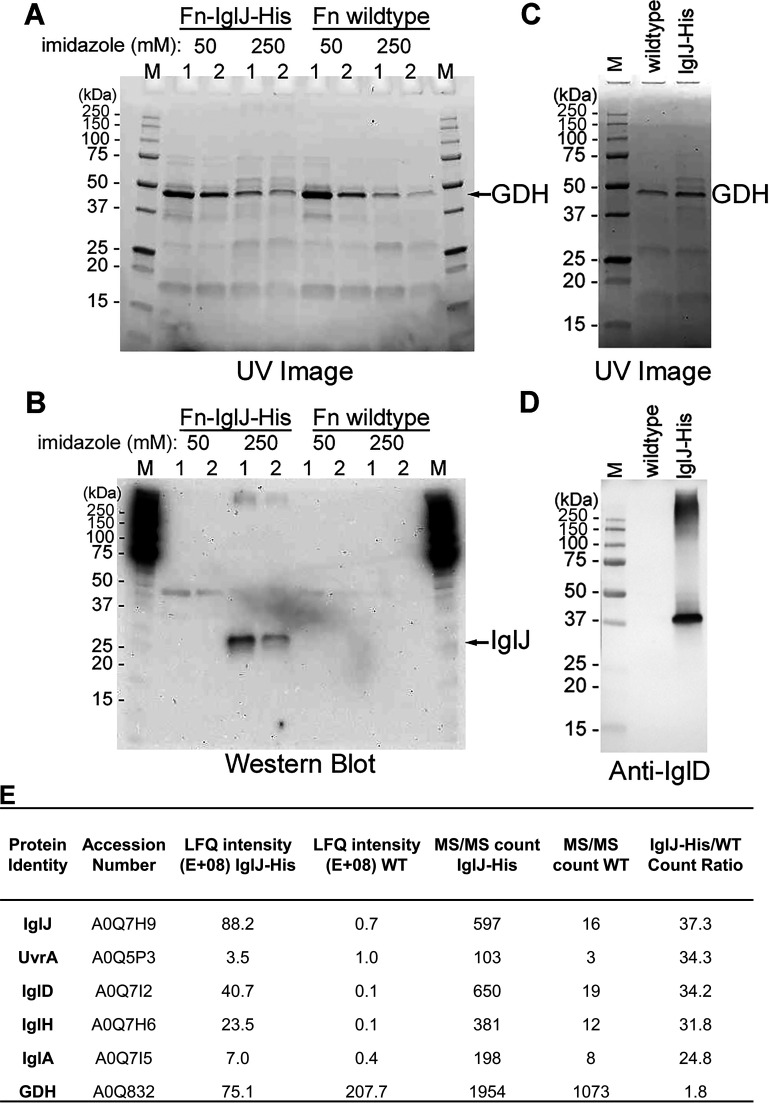
IglJ-His pulls down IglD. Wild-type (WT) Fn or Fn expressing IglJ-His were grown in high KCl, cross-linked with DSP, lysed, and IglJ-associated proteins enriched by nickel-agarose affinity chromatography. (A) SDS-PAGE gel showing proteins eluted in successive fractions with 50 mM and 250 mM imidazole visualized by stain-free UV imaging. (B) Western immunoblot using anti-His epitope antibody identifies IglJ-His in the Fn IglJ-His sample and not in the WT sample. (C and D) Samples eluted with 250 mM imidazole were further analyzed by SDS-PAGE with stain-free UV imaging of proteins (C) and Western immunoblotting using polyclonal antibodies against IglD (D). (E) MS/MS analysis of IglJ-His and WT pulldown samples.

We set out to identify other *Francisella* baseplate components by affinity pulldown and mass spectrometry. In the canonical T6SS, the baseplate consists of 4 proteins, TssE, TssF, TssG, and TssK, that interact to form a wedge complex with stoichiometry TssK_6_-TssG_1_-TssF_2_-TssE_1_ and 6 wedge complexes assemble to form the baseplate (23). While our structural study demonstrated that IglD is the TssK ortholog, the other components of the *Francisella* T6SS baseplate have not been identified. We hypothesized that the *Francisella* protein IglJ corresponds to the TssG of canonical T6SS based on its small size, absence of another assigned role in the T6SS, and the fact that it is essential for T6SS assembly, phagosome escape, and virulence (35). To test this hypothesis, we replaced the chromosomal *iglJ* gene of Fn with a gene encoding a C-terminal His_18_ epitope tag (IglJ-His) and showed that the strain was capable of T6SS-mediated IglC secretion and intracellular growth (Fig. S6). We then induced the T6SS in recombinant and wild-type (WT) control Fn by growing the bacteria in TSBC with 5% KCl. The bacteria were pelleted, washed to remove free amines, cross-linked with dithiobis succinimidyl propionate (DSP), lysed by sonication in RIPA buffer, and the IglJ-His and its associated proteins enriched by Ni-NTA agarose affinity chromatography. While the main protein pulled down by the Ni-NTA affinity resin with or without His-tagging of IglJ was found by mass spectrometry to be glutamate dehydrogenase (GDH) (Fig. 3A), Western immunoblotting confirmed enrichment of IglJ (Fig. 3B) and demonstrated specific pulldown of IglD (Fig. 3C and D), i.e., IglJ and IglD immunoreactive bands were present in the pulldown samples of the IglJ His-tagged strain, but not that of the nontagged parental strain. The similar band intensities of GDH (Fig. 3A) confirmed comparable loading of the affinity resin with the IglJ His-tagged and nontagged samples.

10.1128/mbio.01277-22.6 FIG S6 F. novicida expressing IglJ-His from the chromosome secretes IglC in TSBC-KCl and replicates intracellularly in THP-1 cells. ELISA results showed the presence of T6SS effector IglC (A) and lack of baseplate component IglD (B) in the supernatant fluid of wild-type (WT) and IglJ-His expressing F. novicida strains growing in TSBC containing 5% KCl. (C) A comparable high IglC/IglD signal ratio to that of the WT indicates that Fn-iglJ-His can secrete IglC in a T6SS-mediated fashion. Bacterial lysate prepared from the WT F. novicida and the medium served as the positive- and negative-controls for the IglC and IglD signals, respectively. (D) F. novicida with *iglJ* deletion was not able to grow in THP-1 cells. In contrast, the strain with *iglJ* replaced by i*glJ-his* multiplied to the same level as the WT. Data shown are mean ± standard deviation of duplicate biological samples (A to C) and triplicate biological samples (D). The experiment was conducted twice with similar results. Download FIG S6, JPG file, 0.1 MB. Copyright © 2022 Liu et al. 2022 Liu et al. https://creativecommons.org/licenses/by/4.0/ This content is distributed under the terms of the Creative Commons Attribution 4.0 International license.

To identify additional proteins interacting with IglJ, we acetone-precipitated the IglJ-His and wild-type samples, digested them with trypsin, and identified and quantified the peptides of the constituent proteins by liquid chromatography with tandem mass spectrometry (LC-MS/MS)-based proteomics analysis. The relative specific enrichment of the proteins was determined by calculating the MS/MS spectral count ratios of proteins identified in the IglJ-His sample versus the wild-type sample (Fig. 3E). Of the 5 proteins with the highest spectral count ratios, 4 were FPI encoded proteins (IglJ, IglD, IglH, and IglA). Of the five proteins, IglJ, IglD, and IglH had the highest absolute spectral counts and the highest label-free quantification (LFQ) intensities in the IglJ His-tagged sample, suggesting these three proteins interact as components of the *Francisella* baseplate wedge complex.

**FIG 4:**
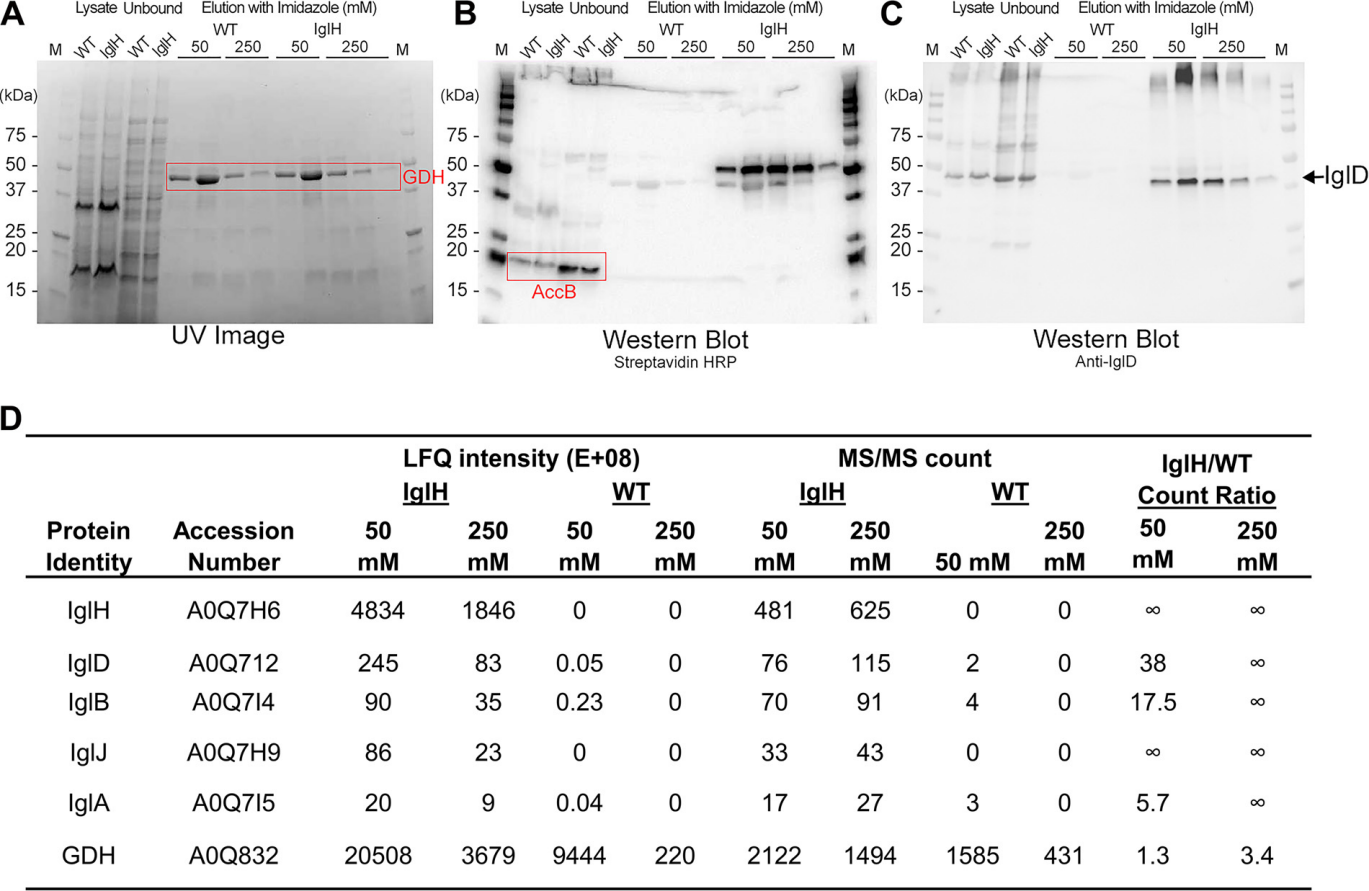
Epitope-tagged IglH pulls down IglD. Wild-type (WT) Fn or Fn expressing IglH with TwinStrep and His-tags were grown in high KCl, cross-linked with DSP, lysed, and IglH-associated proteins enriched by nickel-agarose affinity chromatography. (A) SDS-PAGE gel showing proteins in the lysate, affinity column pass-through (unbound), and eluted in successive fractions with 50 mM and 250 mM imidazole visualized by stain-free UV imaging with glutamate dehydrogenase (GDH) as the most abundant protein eluted by imidazole in both samples. Comparable amounts of GDH demonstrated comparable loading of the affinity resins. (B) Western immunoblot using Streptavidin-peroxidase identifies elution of TwinStrep-tagged IglH in the IglH lanes but not the WT lanes. The endogenous biotinylated protein, AccB (56), was identified at comparable levels in WT and IglH lanes of lysate and unbound samples, again showing comparable loading of the affinity resins. (C) Anti-IglD immunostaining identifies pulldown of IglD in the Fn IglH sample but not in the WT sample. (D) MS/MS analysis of acetone precipitated pellets of the 50 mM and 250 mM eluates of the IglH and WT samples.

To test our hypothesis that IglH is a component of the baseplate wedge complex, we replaced the chromosomal *iglH* gene of Fn with a gene encoding a C-terminal TwinStrep-His_18_ dual epitope tag (IglH-TS-His), induced the T6SS in the recombinant and wild-type control Fn by growing the bacteria in TSBC with 5% KCl, and performed DSP cross-linking and affinity pulldown using Ni-NTA agarose as described above. Western immunoblotting demonstrated that IglD was pulled down in the IglH-TS-His expressing strain but not in the wild-type strain (Fig. 4A). IglJ was not probed in the IglH pulldown immunoblot because IglJ was not epitope-tagged in the strain used and antibody to IglJ was not available to us. However, mass spectrometry-based proteomic analysis of the samples demonstrated that IglJ was pulled down in the IglH-TS-His expressing strain but not in the wild-type strain (Fig. 4B). Together, these results are consistent with the hypothesis that IglD, IglJ, and IglH interact as T6SS baseplate proteins.

### Interrogation of IglD and IglJ interactions by alanine-substitution mutagenesis.

**FIG 5:**
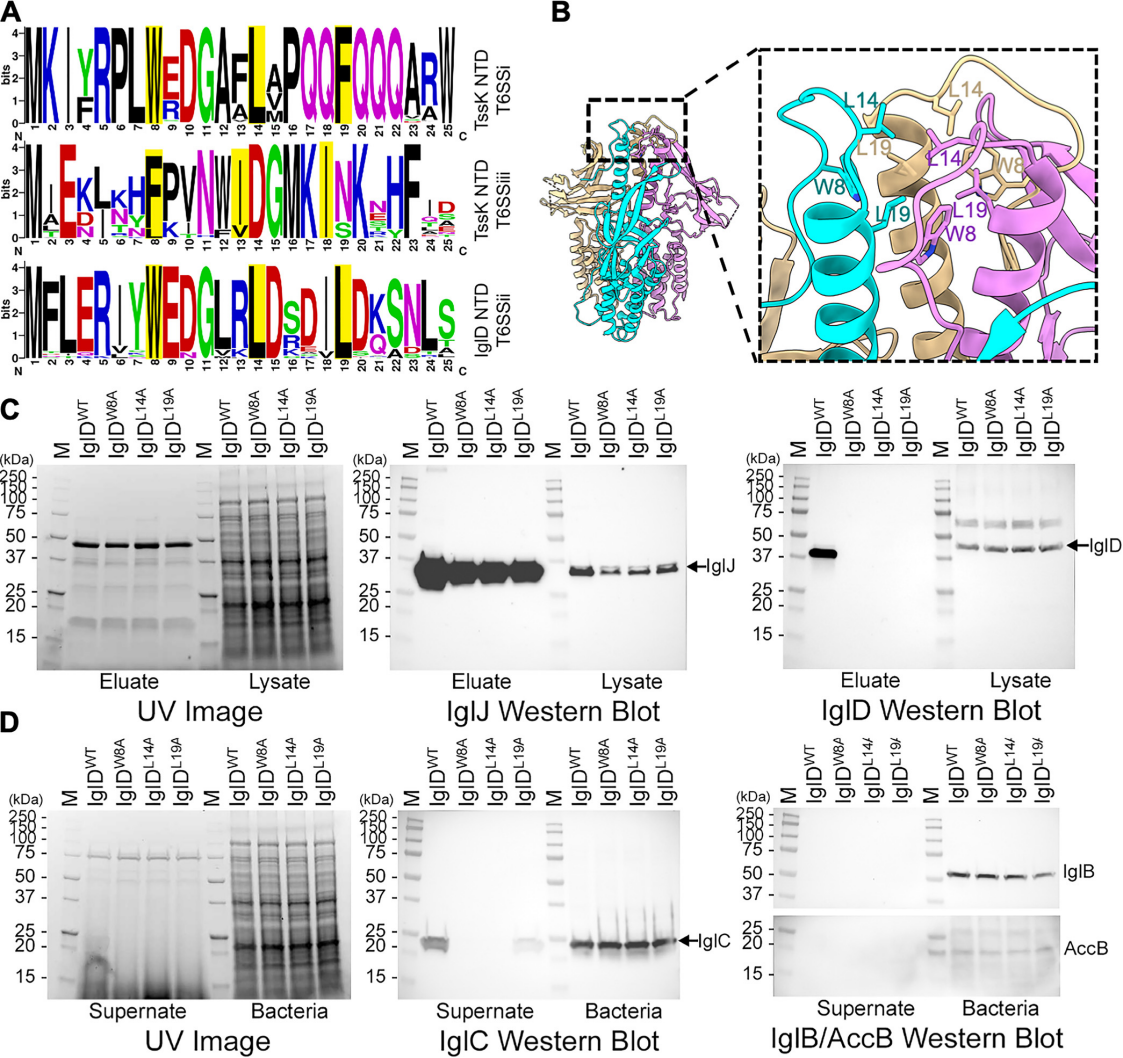
IglD N-terminal domain residues are essential for interaction with IglJ and IglC secretion. (A) Comparison of consensus N-terminal domain sequences of canonical T6SS^i^ TssK, T6SS^iii^ TssK, and *Francisella* (T6SS^ii^) IglD. Conserved residues contributing to the hydrophobic pocket of T6SS^i^ and T6SS^iii^ TssK and T6SS^ii^ IglD are highlighted. (B) Key residues (W8, L14, L19) consistent with TssK-NTD in the IglD structure are shown in stick presentation. Individual protomers of the IglD homotrimer are colored cyan, magenta, and tan. (C) Alanine substitution of IglD NTD residues W8A, L14A, or L19A abolishes the IglJ pulldown of IglD. Stain-free UV image shows comparable lane loading. IglJ-FLAG Western blot shows the capture of IglJ in parental and Ala-substitution strains. IglD Western blot shows pulldown of IglD only in the parental strain. (D) IglD NTD Ala substitution mutants are defective for IglC secretion. Stain-free UV image shows comparable loading of lanes. IglC Western blot shows secretion of IglC into culture supernatant fluid only by the parental strain and comparable levels of IglC in the lysates of all strains. IglB/AccB Western blot shows the absence of leakage of cytosolic proteins IglB or AccB into the culture supernatant fluid.

**FIG 6:**
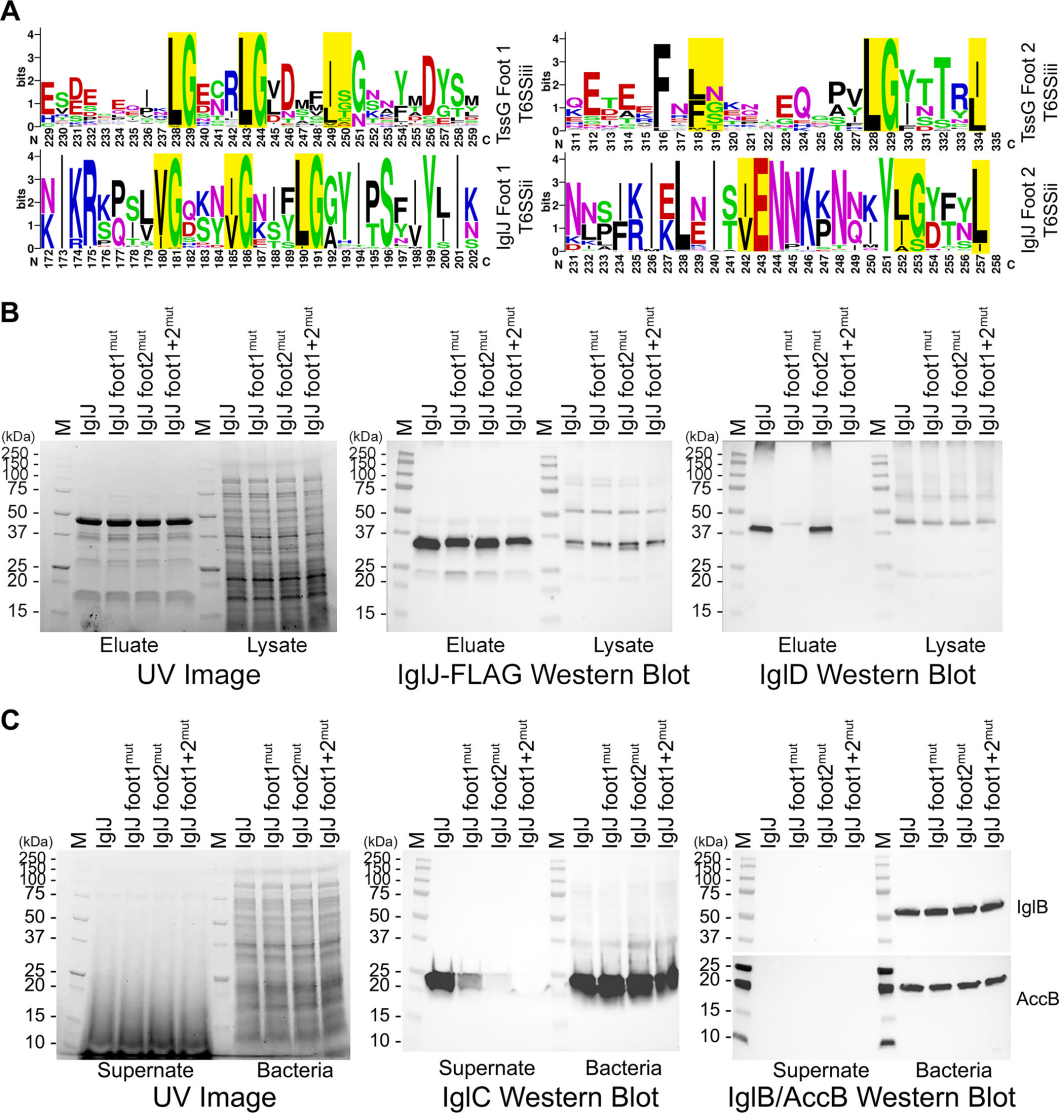
Alanine substitutions in the C terminus of IglJ disrupt pulldown of IglD and decrease IglC secretion. (A) Comparison of consensus sequences of putative T6SS^iii^ TssG and T6SS^ii^ IglJ foot1 and foot2. Putative LG repeat positions of T6SS^iii^ TssG feet (A, top lines) and corresponding sequences conserved in *Francisella* species (A, bottom lines) are highlighted. (B) Alanine substitution of the three hydrophobic residues of IglJ foot1 (I180, V185, L190) abolishes IglJ pulldown of IglD. Stain-free UV image shows comparable lane loading. IglJ-FLAG Western blot shows the capture of IglJ in parental and Ala-substitution strains. IglD Western blot shows pulldown of IglD in the parental strain and the strain with IglJ foot2 substitutions (V242, E243, L252, L257), but not the strains with alanine substitutions in IgJ foot1 or both foot1 and foot2. (C) Impact of alanine substitutions on IglC secretion. Stain-free UV image shows comparable loading of lanes. IglC Western blot shows that alanine substitution of foot1 markedly decreases IglC secretion, substitution in foot2 even more dramatically decreases IglC secretion, and substitution in both feet abolishes IglC secretion. Western blot of the bacterial lysates shows comparable levels of IglC in all of the strains. IglB/AccB Western blot shows the absence of leakage of cytosolic proteins IglB or AccB into the culture supernatant fluid.

**FIG 7:**
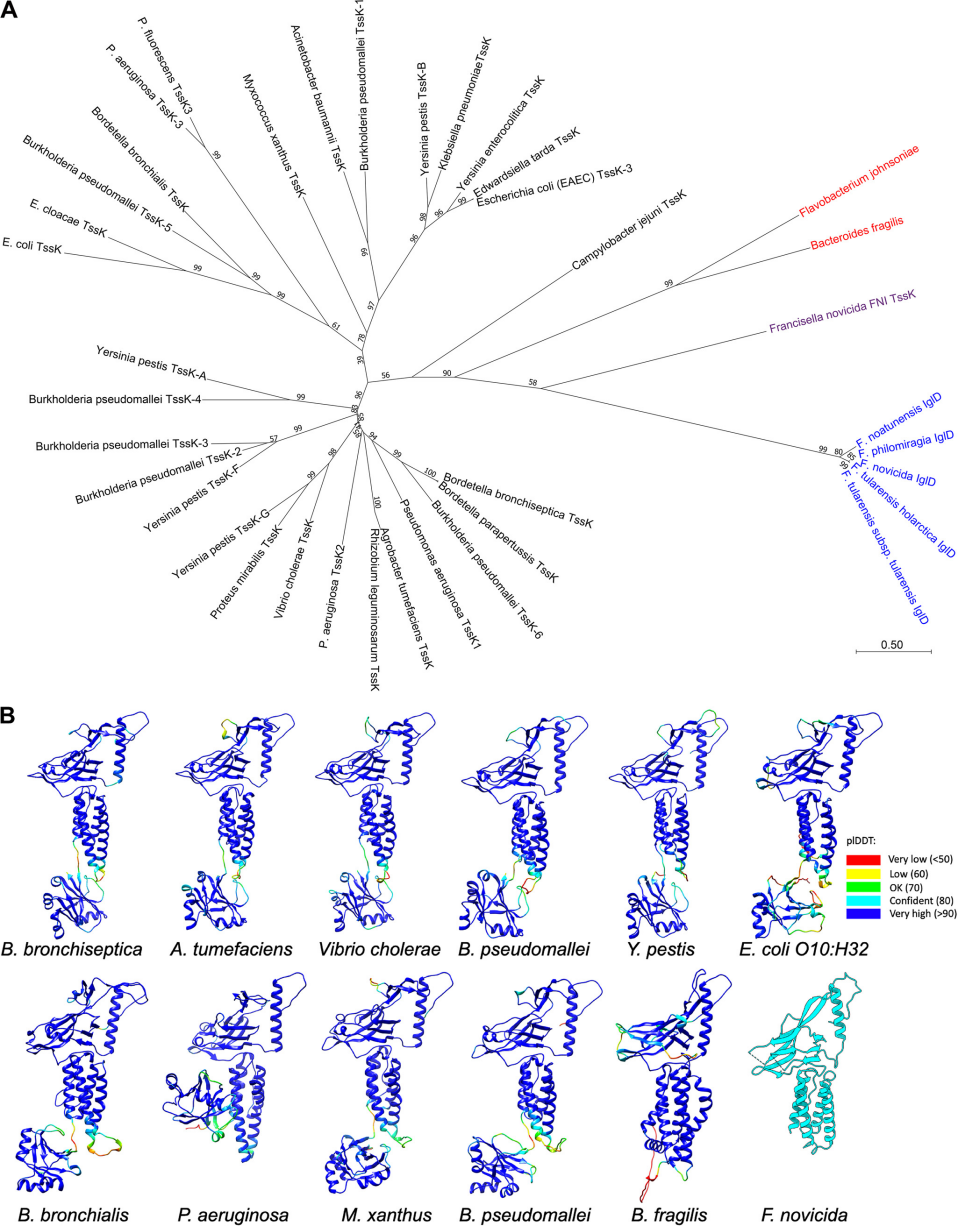
Phylogenetic relationships of IglD and TssK as inferred by using the Maximum Likelihood method and JTT matrix-based model (37). (A) T6SS^i^ are shown in black; T6SS^ii^ (*Francisella*) are shown in blue, and T6SS^iii^ are shown in red. The tree with the highest log likelihood (-29351.96) is shown. The percentage of trees in which the associated taxa clustered together is shown next to the branches. Initial tree(s) for the heuristic search were obtained automatically by applying Neighbor-Join and BioNJ algorithms to a matrix of pairwise distances estimated using the JTT model, and then selecting the topology with a superior log likelihood value. The tree is drawn to scale, with branch lengths measured in the number of substitutions per site. This analysis involved 38 amino acid sequences. Evolutionary analyses were conducted in MEGA11 (39). (B) AlphaFold2 prediction results for TssK/IglD from representative species in (A). The source organism is labeled below each structure. The AlphaFold2 prediction models are colored according to pLDDT confidence. The last one is the Fn IglD structure solved in this study.

Next, we explored how IglD and IglJ interact and what are the structural homologs of IglJ and IglH in canonical T6SS. While BLAST searches of IglJ and IglH yield no significant sequence similarity with any non-*Francisella* proteins, their size and predicted secondary structures suggest that IglJ and IglH correspond to baseplate proteins TssG and TssF, respectively, of canonical T6SS. In the cryoEM structure of the enteroaggregative Escherichia coli (EAEC) T6SS baseplate, TssG has two loops (“feet”) extending from its C-terminal domain and each loop has three conserved hydrophobic residues in a triangular array, with the hydrophobic side chains of each loop projecting into a hydrophobic pocket formed by the N-terminal domains of a TssK trimer (36). Cherrak et al. (37) also identified a conserved sequence in the N-terminal domain of canonical TssK of EAEC corresponding to residues forming the hydrophobic pocket of the TssK trimer and further described each of the two TssG C-terminal feet as having a conserved pattern of three “LG repeats”, wherein “LG” refers to a hydrophobic residue followed by a residue with either a small (e.g., glycine, serine) or basic (e.g., lysine, arginine) side chain. Sequence alignment of TssGs of other T6SS^i^ revealed deviations from the “LG” repeat motif, such as the presence of acidic residues, rather than small or basic residues, in some “LG” repeats of TssG of Klebsiella pneumoniae, Vibrio cholerae, and Yersinia pseudotuberculosis (36). Whereas the basic side chains of EAEC TssG “LG” repeat residues form salt bridges with acidic residues above the N-terminal domain (NTD) hydrophobic pocket of EAEC TssK (36), the acidic residues in the “LG” repeat of these “outliers” may also form salt bridges with basic residues above the NTD of their corresponding TssKs. It is noteworthy that the N terminus of IglD is largely consistent with the NTD consensus sequence of canonical T6SS^i^ TssK (Fig. 5A), including the pivotal residues (W8, L14, L19) that contribute to the hydrophobic pocket (Fig. 5B and Fig. S7) interacting with LG repeats of the TssG feet. We have also identified conserved sequences within predicted loop structures near the C terminus of IglJ (i.e., the putative “feet”) similar to the TssG “LG repeat” motif described by Cherrak et al. (37) that could interact with the hydrophobic pocket in the NTD of IglD, matching the TssK–TssG interactions of canonical T6SS (Fig. 6A). As shown in Fig. 7A, *Francisella* IglD is phylogenetically closer to the T6SS^iii^ TssKs of *Flavobacteria* and *Bacteroidales*, whose NTD has conserved residues similar to those of canonical TssK and *Francisella* IglD (Fig. 5A). Alignment of canonical TssG sequences (T6SS^i^) with those of *Flavobacteria* and *Bacteroidales* TssG (T6SS^iii^) revealed that, whereas the last “LG” repeat of canonical TssG was typically about 40 residues from the C terminus, the last “LG” repeat of T6SS^iii^ TssG was at the very C terminus, with the hydrophobic residue being the last residue (Fig. 6A). We identified similar “LG” repeat sequences in loop regions of the C terminus of *Francisella* IglJ, with the final hydrophobic residue of the last “LG” repeat usually being the last residue of the sequence (Fig. 6A). While the first putative foot of IglJ is entirely within a loop region, the first “LG” repeat (V242, E243) of the second foot is predicted to be the last two residues of β-strand (Fig. S8F). However, the remainder of the second foot (residues 244 to 257) is predicted to be a loop (Fig. S8F), that would allow the hydrophobic side chains of the “LG” positions to form a triangular array for insertion into the IglD NTD hydrophobic pocket. Residue 243 (highly conserved glutamate) is acidic (Fig. 6A), rather than basic, but its sidechain may form a salt bridge with conserved basic residue 13 (arginine or lysine) above the hydrophobic pocket of the IglD NTD (Fig. 5A and Fig. S7). All the alignments and predictions indicate that IglJ functions as the TssG homolog of EAEC T6SS to interact with IglD NTD hydrophobic pocket by two feet.

10.1128/mbio.01277-22.7 FIG S7 Zoom-in top view of IglD NTD. (A) Surface representation with positive, neutral, and negative electrostatic potentials indicated in blue, white, and red, respectively. (B) The same view as (A), with hydrophilic, neutral, and hydrophobic areas, indicated in cyan, white, and gold, respectively. Key residues in each IglD protomer are labeled and colored as cyan, magenta, and yellow. The three black circles indicate three hydrophobic patches that IglJ feet may interact with. Download FIG S7, JPG file, 0.4 MB. Copyright © 2022 Liu et al. 2022 Liu et al. https://creativecommons.org/licenses/by/4.0/ This content is distributed under the terms of the Creative Commons Attribution 4.0 International license.

10.1128/mbio.01277-22.8 FIG S8 (A) EAEC TssG structure (PDB accession no. 6GJ1; Chain C). (B) AlphaFold2 prediction of IglJ, colored according to pLDDT. (C) I-tasser prediction of IglJ using EAEC TssG (PDB accession no. 6GIY; Chain C) as the template. (D) EAEC TssF structure (PDB accession no. 6GJ1; Chain A). (E) AlphaFold2 prediction of IglH, colored according to pLDDT. (F) Sequence alignment of EAEC TssG, *F. marina* TssG (Fm TssG), and F. novicida IglJ (Fn IglJ). The sequence alignment was done with Clustal Omega and displayed with ESPRipt 3. Secondary structures of EAEC TssG (PDB 6GJ1) are shown on top. Secondary structures labeled at the bottom were done according to the Jpred4 predictions of Fn IglJ helix and strand when the Jpred4 predictions were of high confidence (>7). Foot1 and Foot2 locations are indicated. The “LG” repeats or putative “LG” repeats are highlighted. Download FIG S8, JPG file, 1.5 MB. Copyright © 2022 Liu et al. 2022 Liu et al. https://creativecommons.org/licenses/by/4.0/ This content is distributed under the terms of the Creative Commons Attribution 4.0 International license.

To test the hypothesis that IglJ interacts with IglD NTD by two feet, we carried out a pulldown assay with alanine substitutions on IglJ and IglD. In EAEC, mutation of any of the highly conserved residues that contribute to the TssK NTD hydrophobic pocket (W8, L14, F19) leads to a loss of TssG–TssK interaction and T6SS function (37). *Francisella* IglD has highly conserved NTD residues in the same positions (Fig. 5A and B), and we have found that replacing any one of these three residues with alanine (W8A, L14A, or L19A) abolishes the capacity of IglJ to pulldown IglD (Fig. 5C) and abolishes T6SS IglC secretion (Fig. 5D). In the case of EAEC, deletion of either foot1 or foot2 of TssG also blocks TssK pulldown and T6SS biological function (37). However, we found that deletion of either the putative foot2 region of IglJ or the deletion of both putative feet resulted in undetectable IglJ expression, as well as loss of IglC secretion (unpublished data). Therefore, we examined the impact of alanine substitutions in putative IglJ foot1 (I180A, V185A, L190A) and foot 2 (V242A, E243A, L252A, L257A). In foot2 we substituted alanine both for the hydrophobic residue and the highly conserved E243 to eliminate the potential salt bridge interaction. We found that the 4 alanine substitutions in foot2 did not block the IglJ pulldown of IglD (Fig. 6B) and that the three alanine substitutions in foot1 or the combined foot1 and foot2 alanine substitutions were sufficient to block IglJ pulldown of IglD (Fig. 6B). We observed marked impairment of IglC secretion by F. novicida with the 3 alanine substitutions in IglJ foot1, even greater impairment of IglC secretion with the 4 alanine substitutions of foot2, and the abolition of IglC secretion with combined substitutions in both foot1 and foot2 (Fig. 6C). The intracellular growth by these alanine substitution mutants within macrophage-like THP-1 cells paralleled their capacity for IglC secretion (Fig. S9). Specifically, we observed severe impairment of intracellular growth with any of the three alanine substitutions in the IglD NTD, marked impairment in intracellular growth with the 3 IglJ foot1 substitutions, even more marked impairment of growth with the foot2 alanine substitutions, and the combined foot1 and foot2 substitutions were as impaired in growth as a complete *iglJ* deletion (Fig. S9). These data indicate that the putative IglJ foot1 “LG” repeats are necessary and sufficient to enable IglJ-IglD interaction under the stringent conditions of the pulldown assay, which includes high salt (0.3 M NaCl) and 1% Triton X-100, both used to prevent nonspecific pulldown. Under the conditions of the pulldown assay, foot1 may play a stronger role in interacting with IglD because its loop is fixed within two regions of secondary structure, whereas the extreme C-terminal foot2 loop is inherently more flexible and disordered. On the other hand, within the intact bacterium (i.e., under physiological conditions and in the presence of accessory and chaperone proteins), the combination of hydrophobic and salt bridge interactions of foot2 may make it more important in interacting with the IglD NTD. While alanine substitutions in both putative feet of IglJ were required for complete disruption of T6SS IglC secretion, alanine substitutions in any of the three key conserved hydrophobic residues contributing to the IglD NTD hydrophobic pocket were sufficient to disrupt both pulldown and IglC secretion because alteration of the IglD NTD hydrophobic pocket disrupts interactions with both IglJ feet. These data support IglJ and IglD interacting directly by two feet and NTD hydrophobic pocket similar to TssG and TssK interactions, which are essential for T6SS secretion, indicating IglJ is a homolog to TssG.

10.1128/mbio.01277-22.9 FIG S9 Alanine substitutions in the IglD NTD or IglJ putative foot1 or foot2 impair the growth of F. novicida in macrophage-like THP-1 cells. Monolayers of PMA-differentiated THP-1 cells were infected with F. novicida wild-type (WT) or F. novicida expressing IglJ-FLAG-His (IglJ-FH) from the chromosome with or without alanine substitution mutations in the IglD NTD or IglJ foot1 and/or foot2 or with deletion of the *iglJ* gene (ΔIglJ). Numbers of F. novicida in the monolayers were determined at 1, 5, and 22 h after infection. All strains showed similar levels of bacteria associated with the monolayer at 1 h postinfection. F. novicida with alanine substitutions in the IglD NTD (IglD^W8A^, IglD^L14A^, IglD^L19A^) showed a 1.5 to 2 log decrease in intracellular growth at 22 h. F. novicida with alanine substitutions in IglJ putative foot1 or foot2 showed a 0.5-log and 1-log decrease in intracellular growth at 22 h. F. novicida with alanine substitutions in both foot1 and foot2 of IglJ showed more than 2-log impairment in intracellular growth; i.e., growth comparable to deletion of *iglJ*. Download FIG S9, JPG file, 0.2 MB. Copyright © 2022 Liu et al. 2022 Liu et al. https://creativecommons.org/licenses/by/4.0/ This content is distributed under the terms of the Creative Commons Attribution 4.0 International license.

### Phylogenetic analysis shows that IglD is genetically remote from the TssK orthologs in other T6SSs.

In canonical T6SS, TssK is situated at the interface between the baseplate and the membrane complex of the T6SS. Its N-terminal domain interacts with the TssG of the baseplate and its flexible C-terminal domain interacts with the membrane complex (23). As such, TssK functions as an adaptor between the baseplate and membrane complex (23). Whereas the T6SS baseplate proteins TssF, TssG, and TssE have structural orthologs in the baseplates of extracellular contractile injection systems (e.g., myophage, R pyocins, and insecticidal antifeeding prophages [Afps]), TssK and IglD have no corresponding structural counterparts in extracellular contractile injection systems. This is likely attributable to the fact that contractile phages, R pyocins, and Afps have no structure corresponding to a membrane complex.

T6SSs have been divided into 4 classes, with *the Francisella* T6SS being in a class by itself (T6SS^ii^), whereas canonical T6SSs of Gammaproteobacteria – e.g., Vibrio cholerae and Pseudomonas aeruginosa – belong to T6SS^i^; Flavobacterium johnsoniae and Bacteroides fragilis belong to T6SS^iii^, and the Candidatus Amoebophilus asiaticus Afp-like system belongs to T6SS^iv^ and is phylogenetically closer to R pyocins and myophage than to other T6SSs (38). Phylogenetic analysis with MEGA11 (39) using IglD sequences of *Francisella* and TssK sequences of other bacteria confirmed that IglD of F. tularensis is phylogenetically remote from other TssK proteins (Fig. 7A). The AlphaFold2 predictions for TssKs from each main branch of the phylogenetical tree show that TssKs from different species exhibit highly similar folding architecture as *Francisella* IglD whose structure was determined in this study (Fig. 7B), demonstrating again that IglD is a structural homolog to TssK. Fn also has a second T6SS-like gene cluster called the F. novicida island (FNI), which encodes a hypothetical TssK-like protein (40). Phylogenetic analysis places the FNI TssK in between the T6SS^ii^ FPI IglD and the T6SS^iii^ TssKs of F. johnsoniae and B. fragilis (Fig. 7A), suggesting that it may have been acquired by horizontal transfer. The FNI TssK and FPI IglD proteins have sequence homology only at their C-terminal domains (IglD residues 319 to 377) and no significant sequence homology in the regions for which we determined the structure. We did not identify any peptides attributable to the FNI *tssK* gene product in our mass spectrometry studies, and it is not known whether the FNI *tssK* gene is transcribed or functions as part of a T6SS.

## DISCUSSION

**FIG 8:**
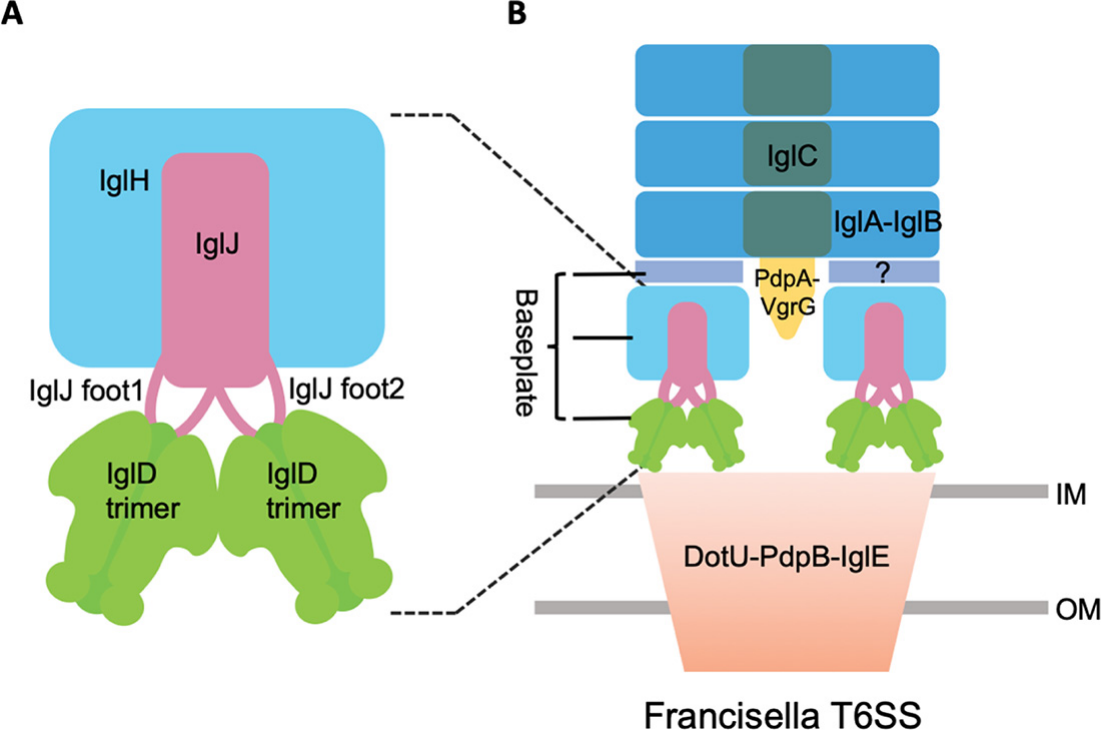
Schematic illustration of Fn T6SS baseplate assembly. (A) IglD trimer (colored green), IglJ (magenta), and IglH (light blue) are components of the Fn T6SS baseplate. IglJ interacts with two IglD homotrimers via foot1 and foot2. (B) The baseplate docks to the DotU-PdpB-IglE membrane complex (orange) and further recruits the tail complex (dark blue and dark green). The question mark within the baseplate indicates the unknown component(s). In particular, the *Francisella* sheath initiator protein, i.e., the TssE ortholog, has not been identified. OM, outer membrane; IM, inner membrane.

In this study, we show that *Francisella* IglD forms homotrimers that are structural orthologs of baseplate protein TssK of canonical T6SS. We show by cross-linking affinity pulldown studies that IglD interacts with IglJ and IglH, and we propose that these proteins function as orthologs of canonical TssG and TssF in the *Francisella* T6SS baseplate (Fig. 8). We used AlphaFold2 to predict the IglH and IglJ structures based on their sequences (Fig. S8). The structure of IglH predicted (Fig. S8E) by AlphaFold2 aligned well with the published structure of TssF (RMSD = 6.2 Å, TM-align (41) score of 0.55). On the other hand, the AlphaFold2 predicted structure of IglJ (Fig. S8B) was very dissimilar to the published structure of TssG while a model of IglJ predicted by I-tasser using TssG as a template (Fig. S8C) showed high similarity with TssG. Additional structural studies will be required to confirm our hypothesis that IgJ and IglH are the *Francisella* orthologs of TssG and TssF.

Each wedge of the canonical T6SS baseplate also contains a fourth protein, TssE, a sheath initiator protein that interacts via a β-strand with the handshake domain of the sheath subunits closest to the baseplate. The *Francisella* TssE ortholog has not yet been identified and the *Francisella* genome lacks any protein with significant homology with canonical TssE.

Elucidation of the structure and composition of the *Francisella* baseplate will be useful in devising strategies to prevent and treat infections caused by F. tularensis. In this regard, it is noteworthy that Cherrak et al. (37) developed a biomimetic cyclic peptide that blocks the interaction of TssG feet with the highly conserved N-terminal domain hydrophobic pocket of TssK, thus blocking T6SS assembly and virulence. Because we showed that the *Francisella* N-terminal domain has both sequence and structural homology with canonical TssK and that mutation of any of the conserved hydrophobic residues of the NTD leads to a loss of IglJ-IglD interaction and a loss of T6SS secretion, a similar strategy will likely be effective for *Francisella*.

## MATERIALS AND METHODS

### Purification of IglD.

Fn expressing N-terminally 3× FLAG-tagged IglD was grown in trypticase soy broth containing 0.2% l-cysteine (TSBC), 20 μg/mL kanamycin, and 5% KCl, rotating at 150 rpm at 37°C to an optical density (OD) of approximately 2. The bacteria were pelleted by centrifugation at 5000 × *g* for 1 h and resuspended in 100 mL of 50 mM Tris-HCl, pH 8.0, 1 mM EDTA, 1 mM N-ethylmaleimide (NEM), 1 mM phenylmethylsulfonyl fluoride (PMSF), supplemented with 100 μg/mL lysozyme and EDTA-free protease inhibitor (Calbiochem, 1:1000) and benzonase (VWR, 1:10,000). The sample was sonicated with a probe tip sonicator (Cell Disruptor model W-375, Heat Systems Ultrasonics, Plainview, NY) while stirring in an ice bath, clarified by centrifugation at 44,400 × *g* for 23 min, and applied to a Q-Sepharose column (XK-26, 13 cm bed height, GE Healthcare). The column was washed with 50 mM Tris-HCl, eluted with a 0 to 1 M NaCl gradient, and fractions containing FLAG-immunoreactive material were identified by sandwich ELISA in which the ELISA plate (high bind, polystyrene 96-well ELISA plate, Costar, Corning Inc.) was coated for 90 min with rat anti-FLAG monoclonal antibody (Novus Biologicals, 10 μg/mL in PBS), blocked with 1% BSA, incubated with fractions from the column (diluted 1:100 in 50 mM Tris-HCl, pH 7.5, containing 0.15 M NaCl [TBS], 0.1% BSA), washed, incubated with mouse anti-FLAG-peroxidase conjugate (MilliporeSigma, 1:2000), washed, and peroxidase developed with TMB substrate (Thermo Fisher Scientific) according to the manufacturer’s directions. FLAG-immunoreactive fractions were pooled, applied to an anti-FLAG agarose (0.5 mL, Sigma) column, and washed sequentially with (i) 50 mM Tris-HCl containing 0.5 M NaCl, (ii) 50 mM Tris-HCl, pH 7.5, containing 10% glycerol, 10 mM MgCl_2_ and 10 mM ATP, and (iii) TBS. The resin was eluted with 10 mL of 3× FLAG peptide (0.1 mg/mL) in TBS. The eluate was concentrated to 1 mL with a 100,000 MW cutoff spin concentrator (Millipore) and applied to a Sephacryl S200HR gel filtration column that was pre-equilibrated with TBS. UV absorbance at 280 nm of the eluate from the column was monitored with a UV monitor (2238 LKB Uvicord SII). FLAG-immunoreactivity in gel filtration fractions was evaluated by a direct ELISA by diluting aliquots from the column 100-fold with 0.05 M NaHCO_3_, pH 9.6, and adding 0.1 mL/well of a high bind, polystyrene 96-well ELISA plates (Costar, Corning Inc.). After 90 min at room temperature, the wells were blocked with 1% BSA in TBS, washed three times with TBS, and incubated for 60 min at room temperature with horseradish peroxidase (HRP)-conjugated mouse anti-FLAG monoclonal antibody (clone M2, MilliporeSigma) diluted 1:2000 with TBS-1% BSA. The wells were washed three times with TBS, and peroxidase activity was developed with 0.1 mL per well of tetramethylbenzidine (TMB) substrate (Thermo Fisher Scientific) according to the manufacturer’s directions. The reaction was stopped by adding 0.1 mL/well of 2 M H_2_SO_4_, and the absorbance at 450 nm was measured with a microplate reader (iMark, Bio-Rad). The peak fractions containing FLAG-immunoreactive material were pooled and concentrated to 1 mL with a 100,000 MW cutoff spin concentrator (MilliporeSigma).

### CryoEM sample preparation and image collection.

For cryoEM sample preparation, 3 μL of purified IglD protein was applied to glow-discharged Quantifoil R 1.2/1.3 gold grids. The grids were blotted with filter paper and then flash-frozen in liquid ethane using FEI Vitrobot Mark IV (Thermo Fisher Scientific). An FEI TF20 cryoEM instrument was used to optimize the freezing conditions. The best grids were obtained with 60 s glow discharge using air and with the Vitrobot sample chamber set at 8°C temperature, 100% humidity, 6 s blotting time, and 1 blotting force.

CryoEM grids were loaded to a Titan Krios electron microscope (Thermo Fisher Scientific) equipped with a Gatan imaging filter (GIF) Quantum LS and a Gatan K3 Summit direct electron detector. The microscope was operated at 300 kV with the GIF energy-filtering slit width set at 20 eV at superresolution mode, yielding a pixel size of 1.1 Å on the sample level. Movies were recorded with SerialEM at a nominal magnification of 81,000×. Defocus was set to −1.8 to −2.6 μm. The total exposure time for each movie was set to 2 s, fractionated equally into 40 frames, giving a total dosage of ~50 e–/Å2 per movie. A total of 2,880 movies were collected for image processing.

### Single particle cryoEM reconstruction and model building.

The workflow of single-particle analysis is summarized in Fig. S3. Frames in each movie were aligned for drift correction with the GPU-accelerated program MotionCor2 (42), generating two averaged micrographs, one with dose weighting (for particles extraction and final reconstructions) and the other without (used for manually screening, particle picking and defocus determination). The averaged micrographs have a calibrated pixel size of 1.1 Å on the specimen scale. Defocus values of micrographs were determined by CTFFIND4 (43). The following image processing was done using RELION 3.1 (44, 45). A total of 7,479,951 particles were picked initially and extracted with a box size of 180 pixels. After 2D classification, 3,701,614 particles were selected. Some of the 2D classes showed strong features of a 3-fold symmetry (Fig. S2D). Therefore, the C3 symmetry was used in the following 3D classifications and 3D refinements until the symmetry expansion. The selected particles were subjected to 3D classification and 3D refinement generating a construction with a resolution of 3.9 Å. Then skip aligning 3D classification was applied. Three good classes totaling 1,138,462 particles were selected and combined, followed by 3D refinement, then re-extracted from dose-weighted micrographs with a box size of 300 pixels. Another round of 3D local refinement was performed with the re-extracted particles, yielding a final 3.0 Å-resolution reconstruction which was used for model building. Symmetry expansion followed by skip align class3D and local refinement without any symmetry (C1) was applied, resulting in a 3.8 Å-resolution reconstruction with a faint density corresponding to the C-terminal domain. We used a Gaussian filter (width 3) in UCSF Chimera to generate a low-resolution reconstruction to identify it (Fig. S2G). We also performed *de novo* C1 reconstruction and obtained a 4.0 Å reconstruction. As expected, this resolution was worse than that (3.8 Å) of our previous C1 reconstruction. The resolution of the cryoEM map was estimated based on the gold-standard Fourier shell correlation criterion, FSC = 0.143. Data collection and processing statistics are given in Table S1.

10.1128/mbio.01277-22.10 TABLE S1 CryoEM data collection, refinement, and validation statistics. Download Table S1, PDF file, 0.04 MB. Copyright © 2022 Liu et al. 2022 Liu et al. https://creativecommons.org/licenses/by/4.0/ This content is distributed under the terms of the Creative Commons Attribution 4.0 International license.

The atomic model of IglD was built and refined manually in COOT (46). The model was further refined using Phenix (47) in real space with secondary structure, Ramachandran, and rotamer restraints. Refinement statistics of the models are summarized in Table S1. AlphaFold2 predictions were run on google Colab. Figures and movies were generated using UCSF Chimera (48) and ChimeraX (49).

### IglJ-His pulldown, western immunoblotting, and mass spectrometry.

Parental Fn U112 and Fn in which the chromosomal *iglJ* gene was replaced with a gene encoding a C-terminal His_18_ epitope tag were each grown in one L of TSBC with 5% KCl to an optical density of 2. The His-tagged and parental bacteria were processed identically in parallel. The bacteria were pelleted by centrifugation (5000 × *g* for 90 min) and washed three times by centrifugation with 50 mM sodium phosphate, pH 7.5, with 5% KCl to remove residual free amines. The washed pellets were resuspended in 35 mL of 50 mM sodium phosphate buffer, 5% KCl, and then freshly prepared dithiobissuccinimidyl propionate (DSP, Lomant's Reagent, Thermo Fisher Scientific) in DMSO was added to achieve a final concentration of 1% DMSO. The bacteria were cross-linked for 45 min while turning end-over-end at room temperature. The reaction was stopped by the addition of 1 M Tris HCl, pH 7.5, to achieve a final concentration of 50 mM. The bacteria were pelleted by centrifugation at 48,000 × *g* for 30 min and resuspended in 10 mM Tris HCl, pH 8.0, 1% Triton X-100, 0.1% sodium deoxycholate, 0.1% sodium dodecyl sulfate, 0.3 M NaCl, 1 mM PMSF, 1 mM NEM, and EDTA-free protease inhibitor cocktail (1:100, Calbiochem) and benzonase (1:1000). The samples were sonicated with a probe tip sonicator and insoluble material pelleted by centrifugation at 25,000 × *g* for 60 min at 4°C. The clarified lysates were adjusted to contain 10 mM imidazole and rotated overnight with 0.25 mL Ni-NTA agarose that had been equilibrated with the same buffer (Qiagen). The Ni-NTA resins were washed with 10 mM Tris-HCl, 0.3 M NaCl, 1% Triton X-100, 0.1% deoxycholate, and 0.1% SDS containing (RIPA buffer) 10 mM imidazole, and eluted sequentially with 20 mM, 50 mM, and 250 mM imidazole in the same buffer (8 column volumes of each, i.e., 2 mL collected in 1 mL fractions). IglD immunoreactive material and IglJ-His were detected by Western immunoblotting in the 250 mM eluates of the IglJ-His sample but not in the parental sample. Protein in the 250 mM eluates was acetone precipitated by adding 4 volumes of acetone, storing the samples overnight at −20°C, and centrifuging at 10,000 × *g* for 10 min at 4°C. The pellets were washed twice with 4:1 acetone:water at 4°C, air dried for 30 min at room temperature, and stored at −20°C. Further processing was conducted by the UCLA Proteome Research Center. The pellet was resuspended in 8 M urea, 100 mM Tris-HCl, pH 8.5; reduced with 5 mM tris(2-carboxyethyl)phosphine (TCEP); alkylated with 10 mM iodoacetamide, and digested with sequencing-grade trypsin. The peptide mixture was desalted, fractionated on-line using C18 reverse phased chromatography, and analyzed using tandem mass spectrometry on a Q-Exactive mass spectrometer (Thermo Fisher Scientific). Data analysis was performed using IP2 (Integrated Proteomics Applications) against an Fn U112 database (taxid no. 401614) and filtered using a decoy-database estimated false discovery rate of less than 0.01.

### IglH-TwinStrep-His pulldown and western immunoblotting.

Parental Fn U112 and Fn in which the chromosomal *iglH* gene was replaced with a gene encoding a C-terminal TwinStrep-His_18_ dual epitope tag were each grown in 250 mL of TSBC with 5% KCl to an optical density of 2. The epitope-tagged and parental bacteria were processed identically in parallel. The bacteria were pelleted and washed to remove free amines as described above; resuspended in 10 mL of 50 mM sodium phosphate buffer, 5% KCl; cross-linked for 45 min with 1 mM DSP; pelleted; lysed in RIPA buffer, and affinity enriched with Ni-NTA agarose (0.2 mL resin per sample) as described above. IglD immunoreactive material and IglH-TS-His were detected by Western immunoblotting in the 50 mM and 250 mM eluates of the IglH-TS-His sample but not in the nontagged parental sample.

### Gene deletion and epitope tagging.

The 1 to 1.5 kb upstream and downstream neighbor regions flanking the *iglD* or *iglJ* genes were amplified from genomic DNA isolated from Fn U112. The upstream and downstream fragments were joined with overlap extension PCR and cloned into pMP590 for use to generate gene deletion mutants by allelic exchange (19, 50). For the generation of an Fn strain expressing *iglJ-his* in the chromosome, the *iglJ* gene was amplified with a short linker encoding GSGSGSGSGSGS and an expression tag of 18 histidine residues at the 3′-end, and the upstream, *iglJ-his* and downstream fragments were joined with overlap extension PCR and cloned into pMP590. Alanine substitutions of iglD and iglJ were generated using overlapping extension PCR. For episomal expression, *iglD* was amplified from Fn genomic DNA with a FLAG expression tag at the 5′-end and cloned into pFNLTP6 (51) in which the *gro*E promoter was replaced by the *bfr* promoter of F. tularensis subsp. holarctica live vaccine strain. All plasmid constructs were confirmed by nucleotide sequencing.

### T6SS-mediated IglC secretion.

Fn strains were inoculated in TSBC containing 5% KCl at A550 of 0.05 and grown at 37°C, 240 rpm for 14 h. Bacteria were pelleted by centrifugation at 11,000 rpm for 10 min. The supernatant fluid was collected, diluted 32-fold in 50 mM bicarbonate buffer, pH 9.6, and added to a 96-well high binding assay plate (Corning, Kennebunk, ME). After a 2-h incubation, the plate was washed with PBS and blocked with 1% BSA in TBS before incubating with rabbit anti-IglC or IglD primary antibody (1:1000) for 90 min and subsequently HRP-conjugated goat anti-rabbit IglG(H+L) (1:2000) for 1 h at room temperature. The enzymatic reaction was developed by the addition of TMB substrate and stopped with 2 M H_2_SO_4_ solution according to the manufacturer’s directions (Pierce TMB, Thermo Fisher). The absorbance at 450 nm and 570 nm was read using an iMark Microplate Reader (Bio-Rad).

### Intracellular growth measurement.

Human THP-1 monocytic cells (ATCC TIB-202) were differentiated with PMA for 3 days and infected with Fn strains at a multiplicity of infection ratio of 2:1 (bacteria:cell) for 2 h. The infected monolayer was treated with a culture medium containing 10 μg/mL gentamicin for 30 min to kill extracellular bacteria, washed with HBSS, and the last wash was replaced with DMEM containing 10% FBS and 0.1 μg/mL gentamicin. At 1 and 21 h postinfection, the cells were lysed with 1% saponin in PBS for 5 min at 1 h and 21 h postinfection, serially diluted in PBS, and the diluted lysates plated on GCII chocolate agar to determine the number of bacteria in the monolayer.

### Phagosome integrity assessment.

Phagosomal integrity was assessed by a modification of the method of Checroun et al. (52) as previously described (53). THP-1 monocytic cells were differentiated with PMA on coverslips and infected with Fn for 5 h. The infected monolayer was treated with 4% paraformaldehyde in PBS for 1 min followed by 4% paraformaldehyde in 25 mM HEPES-KOH, pH 7.0, 125 mM potassium acetate, 2.5 mM magnesium acetate (KHM) with 15% sucrose, and 50 μg/mL digitonin for 1 min. The monolayer was washed twice with KHM buffer with 15% sucrose and incubated with rabbit anti-F. tularensis or chicken anti-Fn antibody in KHM buffer with 15% sucrose and 0.1% BSA for 15 min at 37°C. The monolayer was washed twice with KHM buffer with 15% sucrose and fixed for 30 min in 4% paraformaldehyde in 0.075 M sodium phosphate. The monolayer was then washed twice with PBS and permeabilized with 1% Triton X-100 in TBS for 30 min. Intracellular Fn was subsequently stained with appropriate primary and secondary antibodies. Cell and bacterial DNA were stained with DAPI. The coverslips were mounted with Prolong gold antifade medium (Life Sciences) and imaged with an Eclipse TE2000 (Nikon) inverted fluorescence microscope equipped with a SPOT camera and software.

### *In silico* analyses.

For phylogenetic tree generation, TssK sequences and IglD sequences of representative bacteria of the various T6SS subtypes (corresponding to the species included in our previously published phylogenetic tree (54)) were downloaded from Uniprot and a phylogenetic tree was generated using the Maximum Likelihood Method by MEGA11 (39).

Consensus sequences of the TssK and IglD NTD were prepared by downloading TssK and IglD sequences from BLAST and aligning the sequences with the MUSCLE multisequence alignment program (https://www.ebi.ac.uk/Tools/msa/muscle/). Outliers were eliminated and consensus sequences were displayed by WebLogo (55).

### Data availability.

The cryoEM density map has been deposited in the Electron Microscopy Data Bank under accession codes EMD-27656. The atomic coordinate has been deposited in the Protein Data Bank under accession code 8DQL.
