# Supplementary figures and images for: Atomic Structure of IglD Demonstrates Its Role as a Component of the Baseplate Complex of the Francisella Type VI Secretion System
Source: mBio. 2022 Aug 29;13(5):e01277-22. doi: 10.1128/mbio.01277-22 (PMC9600919; doi:10.1128/mbio.01277-22)

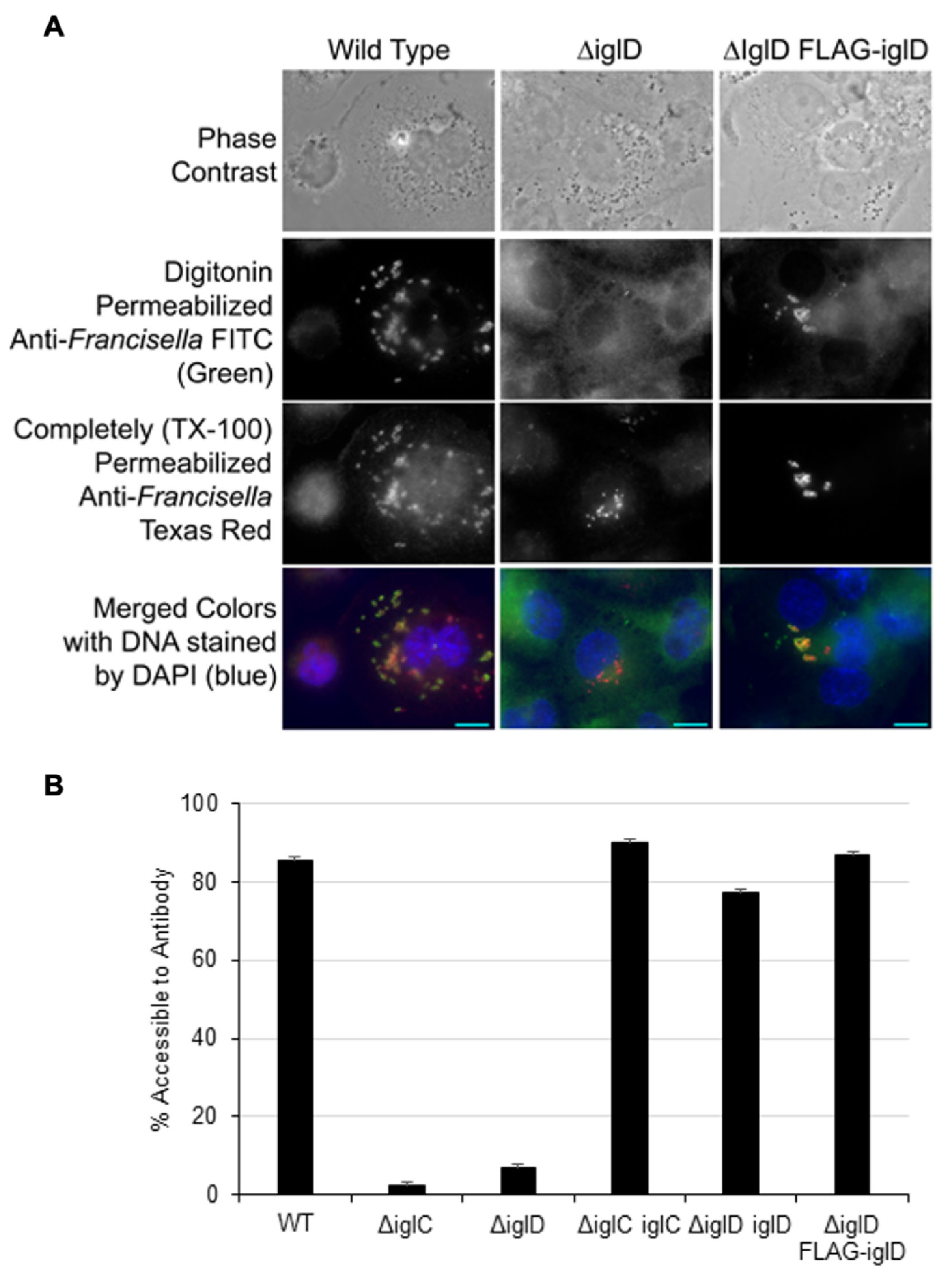

Supplement: FIG S1 [file mbio.01277-22-s0001.jpg]

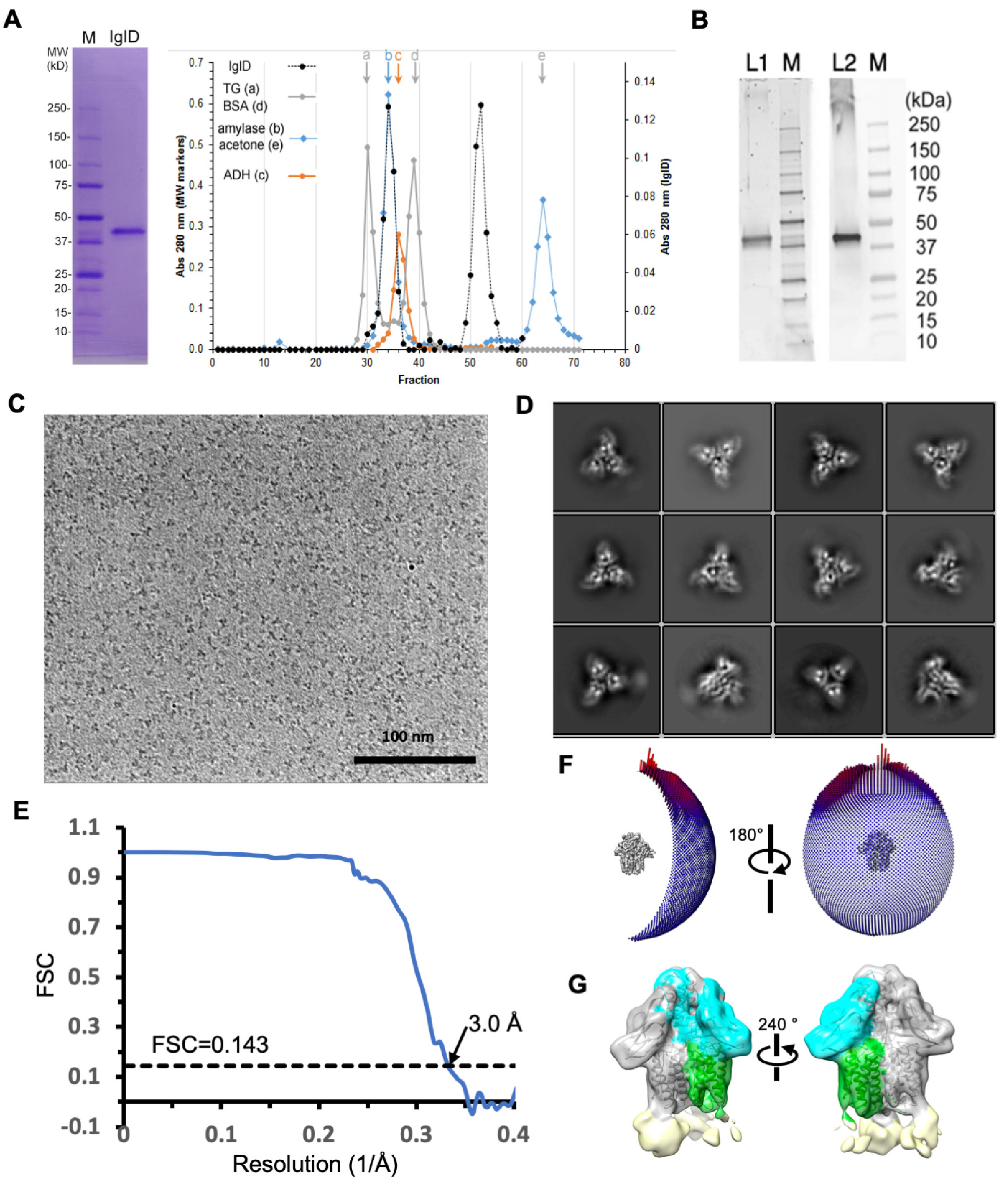

Supplement: FIG S2 [file mbio.01277-22-s0002.jpg]

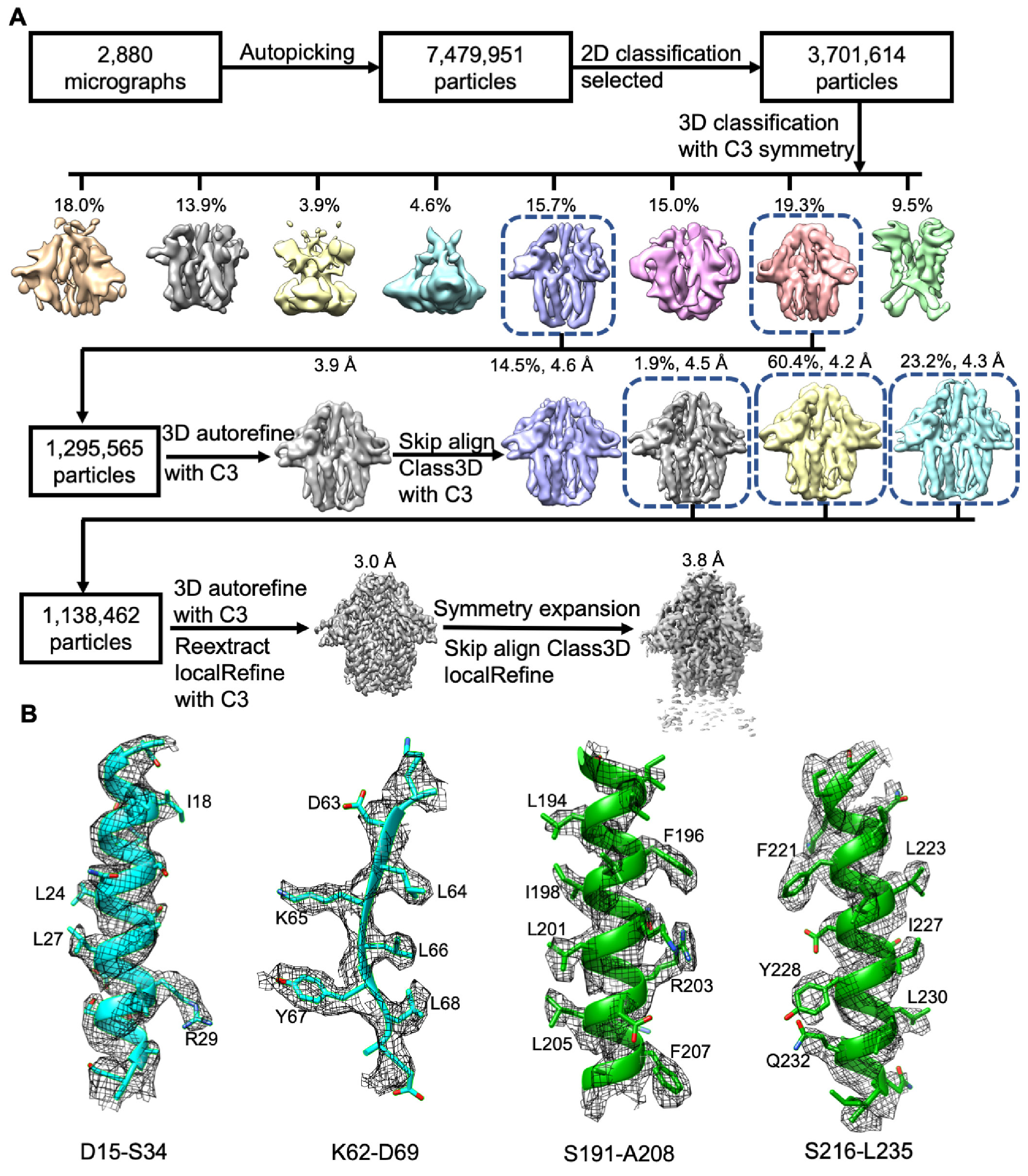

Supplement: FIG S3 [file mbio.01277-22-s0003.jpg]

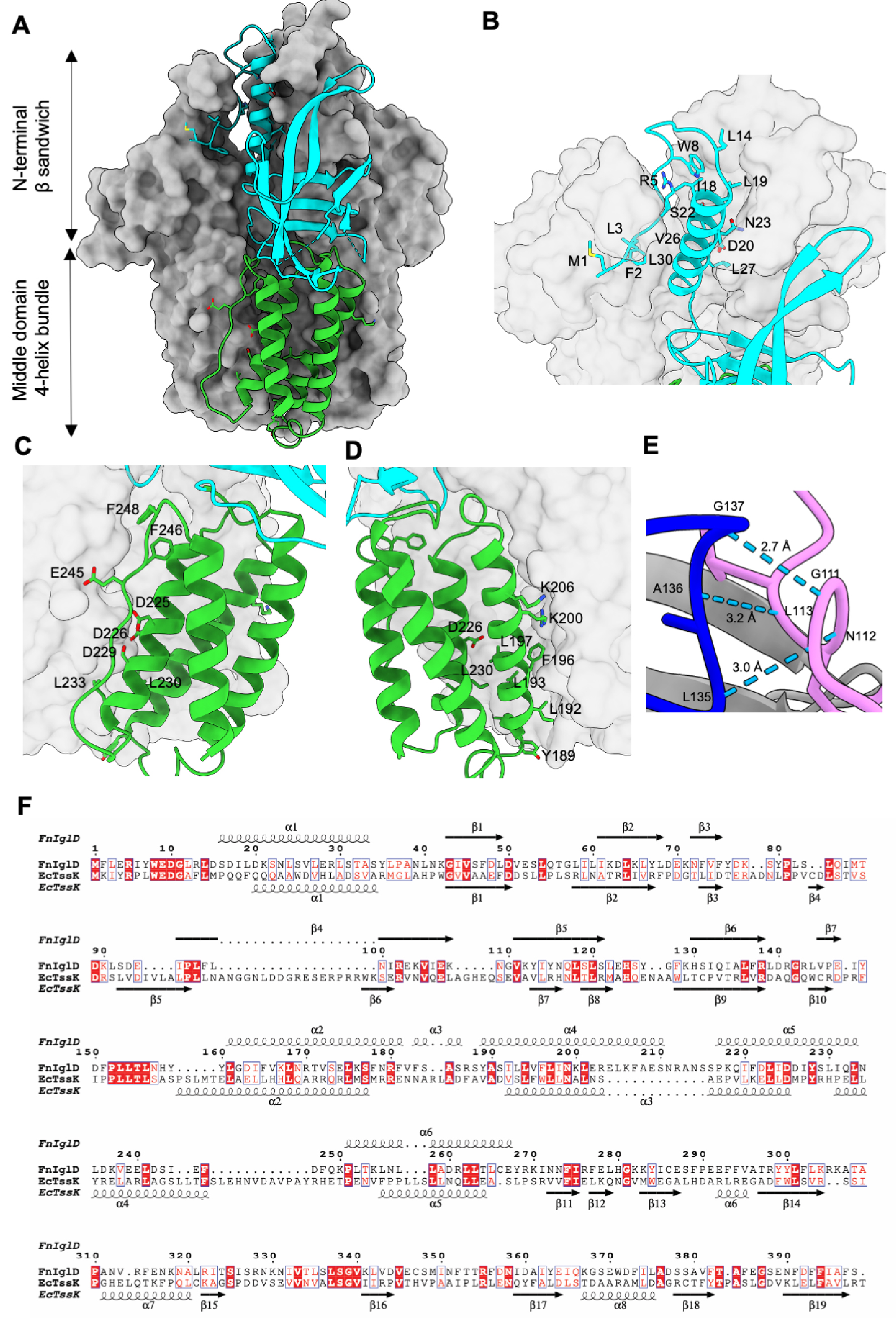

Supplement: FIG S4 [file mbio.01277-22-s0004.jpg]

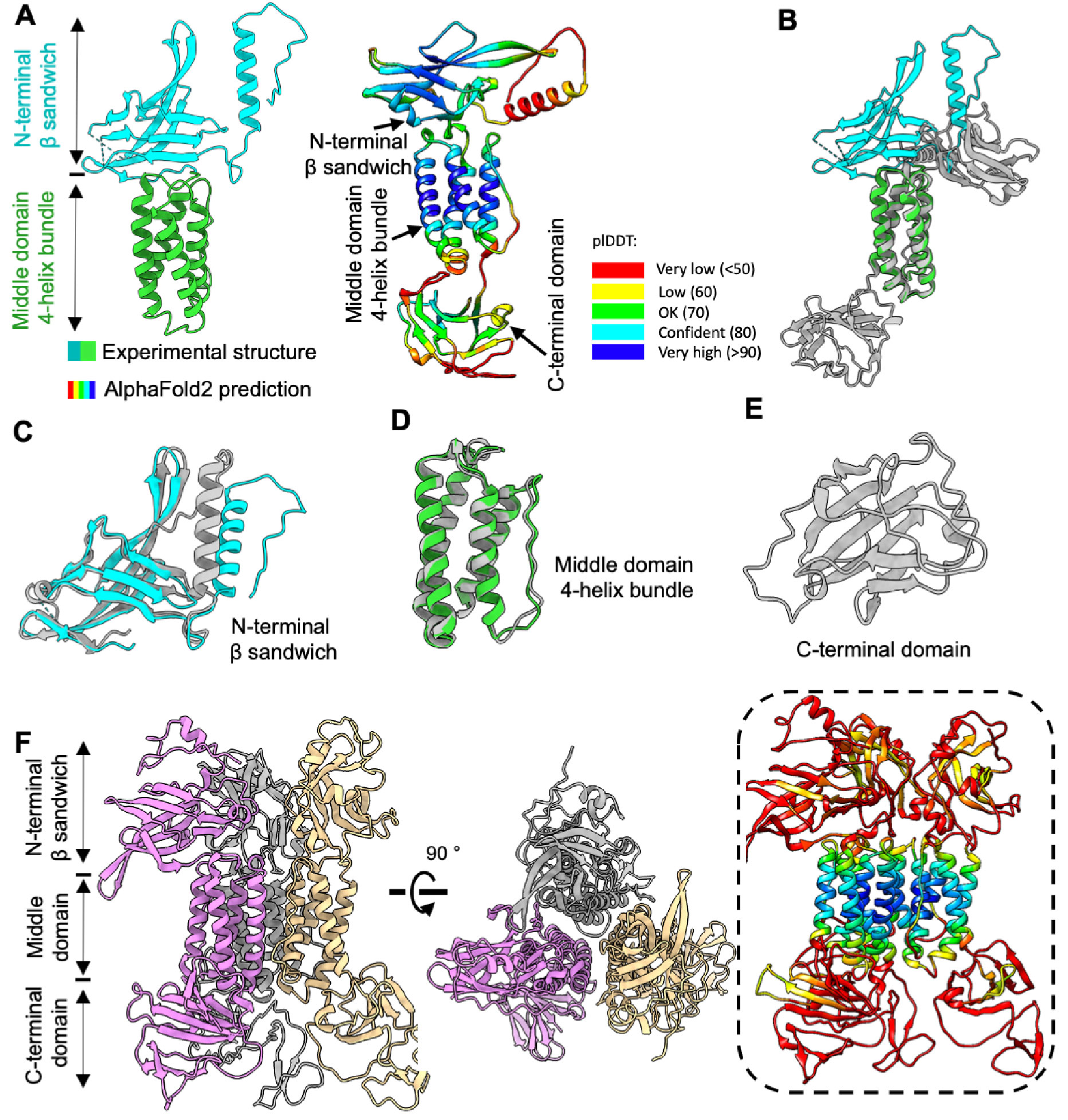

Supplement: FIG S5 [file mbio.01277-22-s0005.jpg]

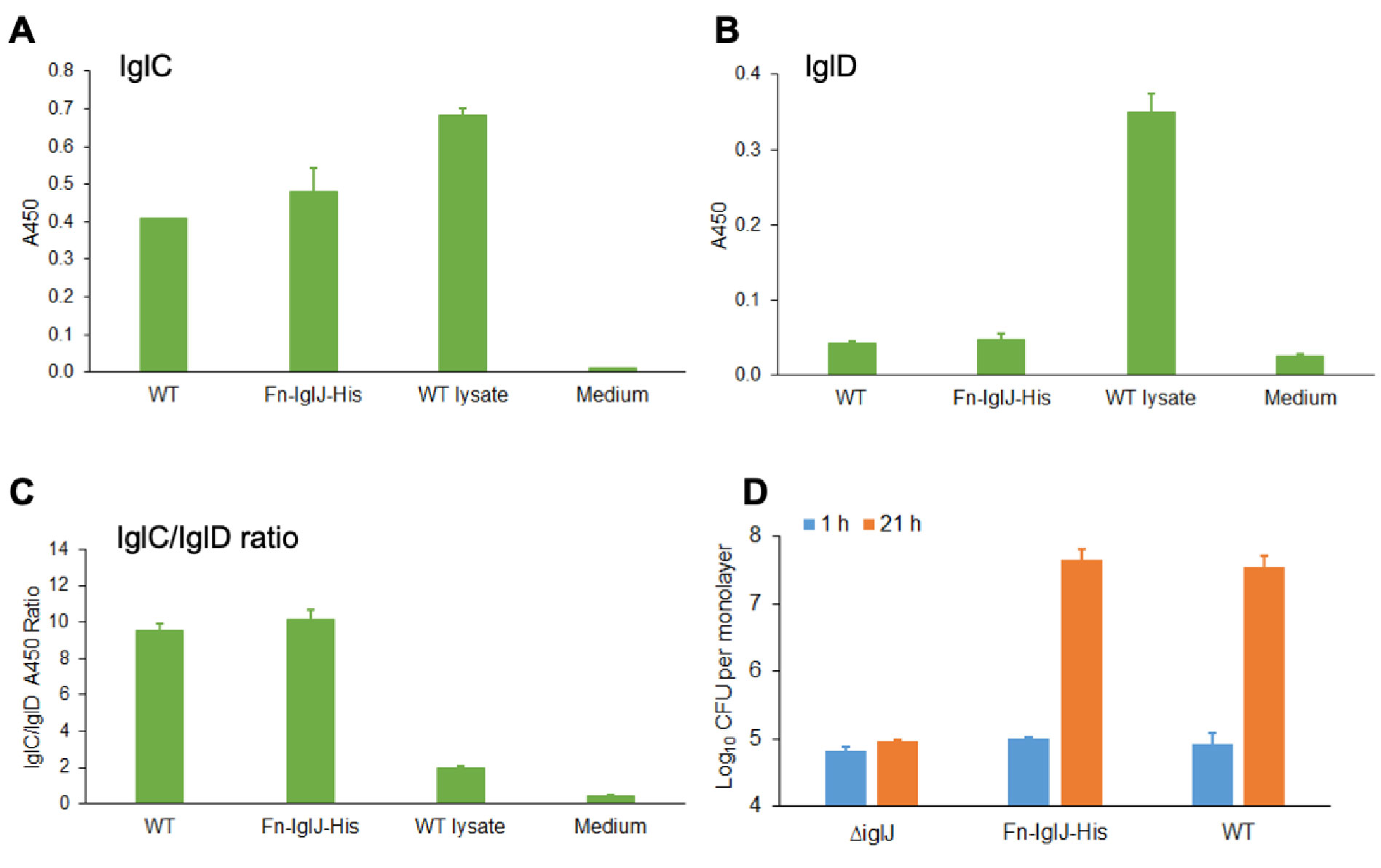

Supplement: FIG S6 [file mbio.01277-22-s0006.jpg]

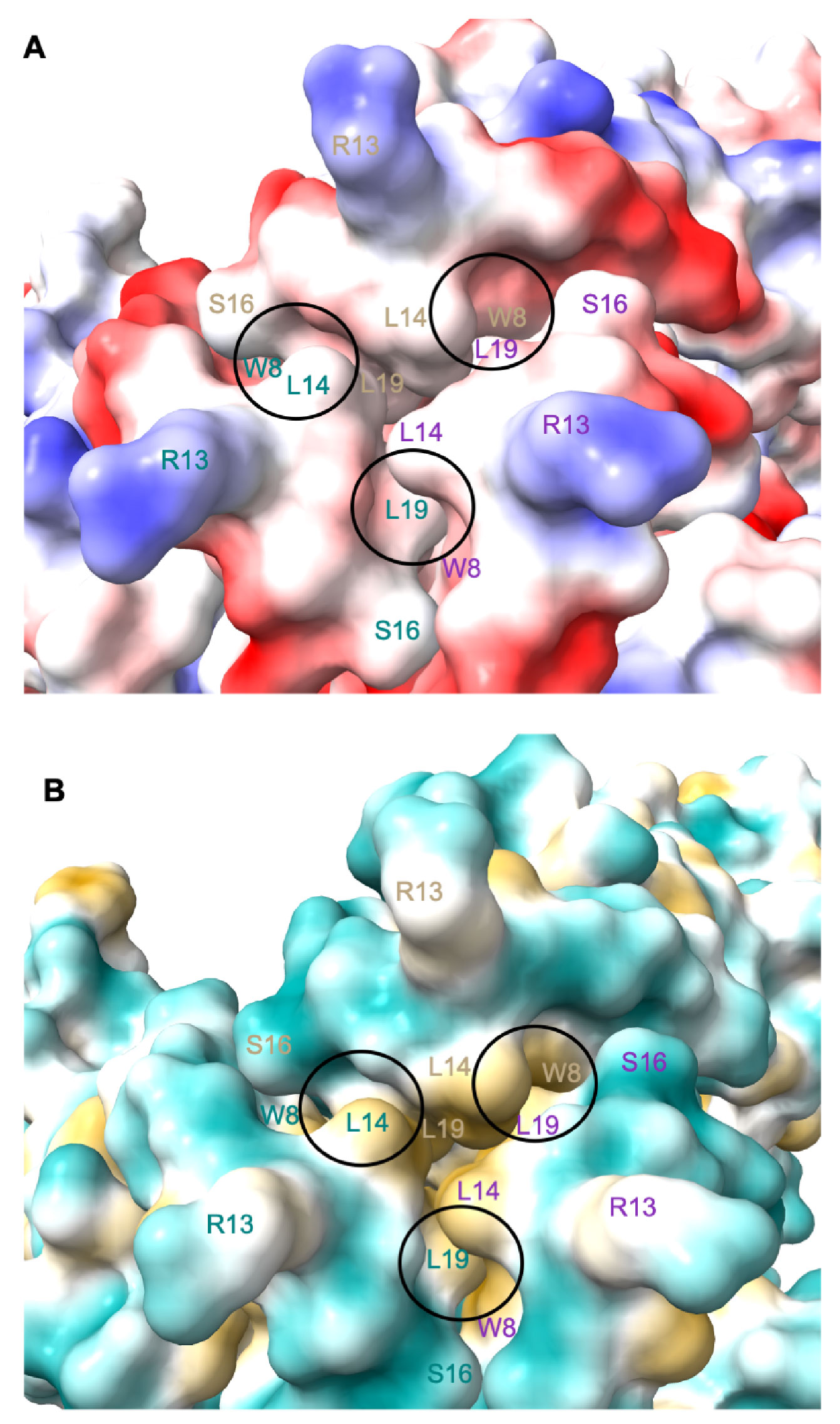

Supplement: FIG S7 [file mbio.01277-22-s0007.jpg]

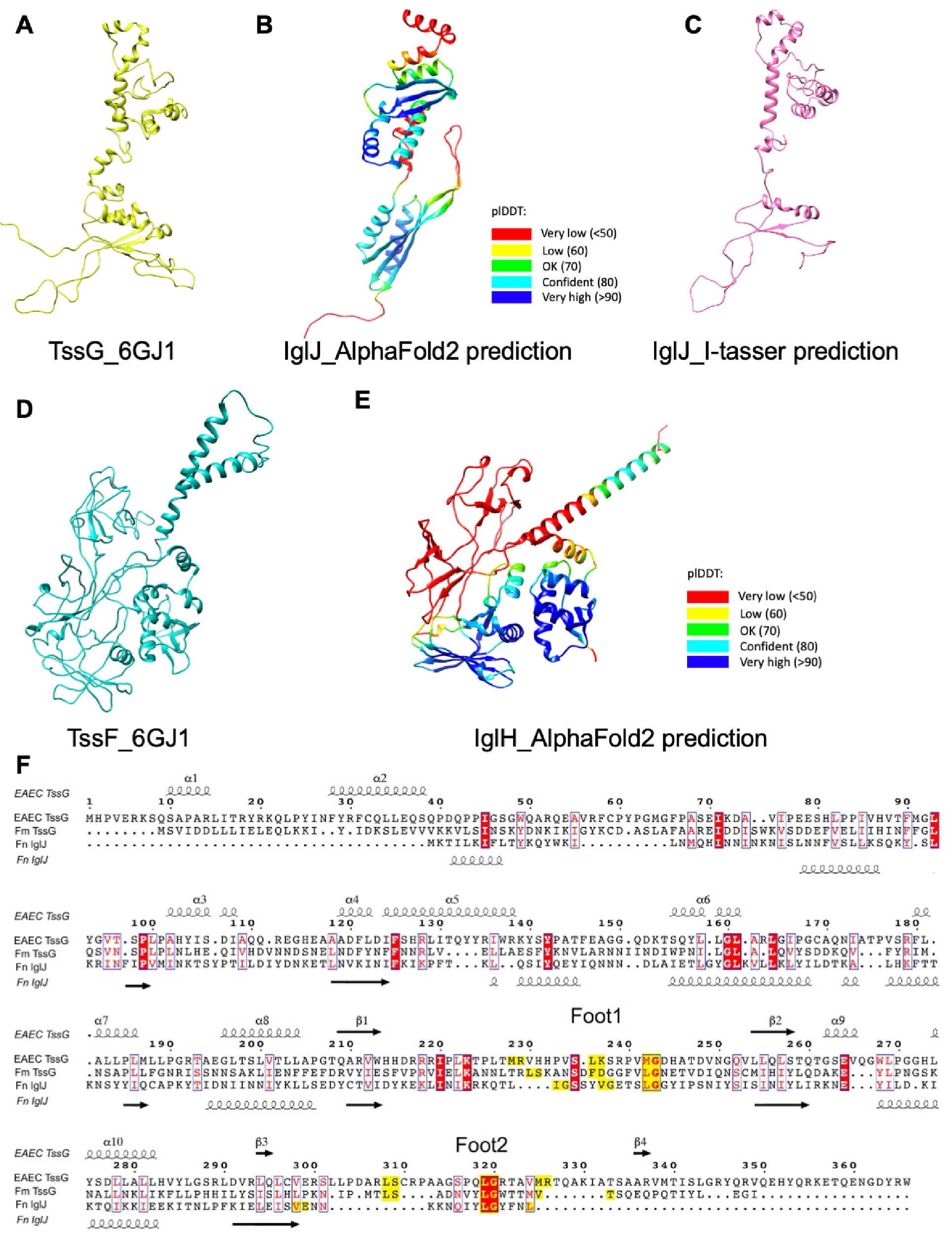

Supplement: FIG S8 [file mbio.01277-22-s0008.jpg]

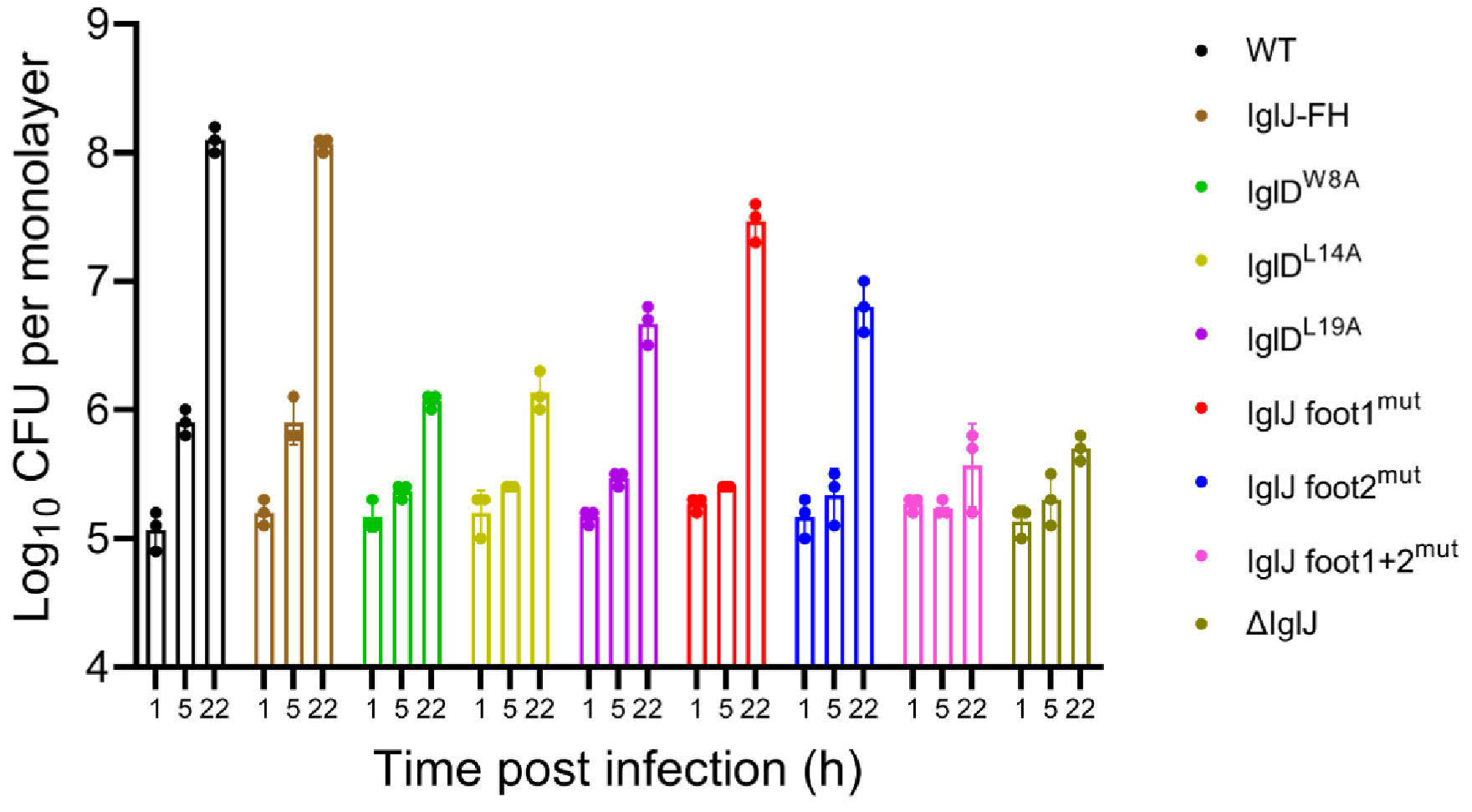

Supplement: FIG S9 [file mbio.01277-22-s0009.jpg]
