# Supplementary material for: Atomic Structure of IglD Demonstrates Its Role as a Component of the Baseplate Complex of the Francisella Type VI Secretion System
Source: mBio. 2022 Aug 29;13(5):e01277-22. doi: 10.1128/mbio.01277-22 (PMC9600919; doi:10.1128/mbio.01277-22)
Supplement: TABLE S1 [file mbio.01277-22-s0010.pdf]

Table S1. CryoEM data collection, refinement and validation statistics

	IglD<br>EMD-27656<br>PDB 8DQL
Data collection and processing	
Magnification	81000
Voltage (kV)	300
Electron exposure ($e^-/Å^2$)	50
Defocus range (μm)	-1.8 to -2.6
Pixel size (Å)	1.1
Symmetry imposed	C3
Particle number	1138462
Map resolution	3.0
FSC threshold	0.143
Refinement	
Map sharpening *B* factor ($Å^2$)	-163
Model composition	
Non-hydrogen atoms	6402
Protein residues	771
Ligand	
B factors ($Å^2$)	
Protein	51.8
Ligand	
R.m.s. deviations	
Bond lengths (Å)	0.002
Bond angle (°)	0.473
Validation	
MolProbity score	1.51
Clashscore	5.1
Poor rotamers (%)	0
Ramachandran plot	
Favored (%)	96.41
Allowed (%)	3.59
Disallowed (%)	0
